# Targeting RBM39 through indisulam induced mis-splicing of mRNA to exert anti-cancer effects in T-cell acute lymphoblastic leukemia

**DOI:** 10.1186/s13046-024-03130-8

**Published:** 2024-07-24

**Authors:** Tongting Ji, Yang Yang, Juanjuan Yu, Hongli Yin, Xinran Chu, Pengju Yang, Ling Xu, Xiaodong Wang, Shaoyan Hu, Yizhen Li, Xiaochen Wu, Wengyuan Liu, Bi Zhou, Wenjuan Wang, Shuqi Zhang, Wei Cheng, Yanling Chen, Lei Shi, Zhiheng Li, Ran Zhuo, Yongping Zhang, Yanfang Tao, Di Wu, Xiaolu Li, Zimu Zhang, Jun-jie Fan, Jian Pan, Jun Lu

**Affiliations:** 1 Children’s Hospital of Soochow University, Suzhou, 215003 China; 2 Institute of Pediatric Research, Children’s Hospital of Soochow University, No.92 Zhongnan Street, SIP, Suzhou, 215003 China; 3 Department of Hematology, Children’s Hospital of Soochow University, No.92 Zhongnan Street, SIP, Suzhou, Jiangsu 215003 China; 4 Department of Pediatric, The Affiliated Hospital of Xuzhou Medical University, Xuzhou, 221000 China; 5 Department of Pediatrics, The Second Affiliated Hospital of Anhui Medical University, No. 678 Furong Road, Hefei City, 230601 China; 6 Department of Pediatric, Suzhou Hospital of AnHui Medical University, Suzhou, 234000 China; 7 Department of Pharmacy, Children’s Hospital of Soochow University, Suzhou, Jiangsu 215025 China; 8 Department of Pediatrics, The First Affiliated Hospital of Wannan Medical College, Wuhu, 241002 China; 9 Department of Medicinal Chemistry, Jiangsu Key Laboratory of Drug Design and Optimization, China Pharmaceutical University, Nanjing, 210009 China; 10 Department of Orthopaedics, Children’s Hospital of Soochow University, Suzhou, 215003 China

**Keywords:** Indisulam, RBM39, T-ALL, RNA splicing, THOC1

## Abstract

**Background:**

Despite the use of targeted therapeutic approaches, T-cell acute lymphoblastic leukemia (T-ALL) is still associated with a high incidence of complications and a poor prognosis. Indisulam (also known as E7070), a newly identified molecular glue compound, has demonstrated increased therapeutic efficacy in several types of cancer through the rapid degradation of RBM39. This study aimed to evaluate the therapeutic potential of indisulam in T-ALL, elucidate its underlying mechanisms and explore the role of the RBM39 gene.

**Methods:**

We verified the anticancer effects of indisulam in both in vivo and in vitro models. Additionally, the construction of RBM39-knockdown cell lines using shRNA confirmed that the malignant phenotype of T-ALL cells was dependent on RBM39. Through RNA sequencing, we identified indisulam-induced splicing anomalies, and proteomic analysis helped pinpoint protein changes caused by the drug. Comprehensive cross-analysis of these findings facilitated the identification of downstream effectors and subsequent validation of their functional roles.

**Results:**

Indisulam has significant antineoplastic effects on T-ALL. It attenuates cell proliferation, promotes apoptosis and interferes with cell cycle progression in vitro while facilitating tumor remission in T-ALL in vivo models. This investigation provides evidence that the downregulation of RBM39 results in the restricted proliferation of T-ALL cells both in vitro and in vivo, suggesting that RBM39 is a potential target for T-ALL treatment. Indisulam’s efficacy is attributed to its ability to induce RBM39 degradation, causing widespread aberrant splicing and abnormal translation of the critical downstream effector protein, THOC1, ultimately leading to protein depletion. Moreover, the presence of DCAF15 is regarded as critical for the effectiveness of indisulam, and its absence negates the ability of indisulam to induce the desired functional alterations.

**Conclusion:**

Our study revealed that indisulam, which targets RBM39 to induce tumor cell apoptosis, is an effective drug for treating T-ALL. Targeting RBM39 through indisulam leads to mis-splicing of pre-mRNAs, resulting in the loss of key effectors such as THOC1.

**Supplementary Information:**

The online version contains supplementary material available at 10.1186/s13046-024-03130-8.

## Background

T-cell acute lymphoblastic leukemia (T-ALL) is a hematologic malignancy that exhibits aggressive characteristics. It is characterized by the abnormal proliferation of immature T cells in the bone marrow and lymphoid tissues [1]. The application of treatment strategies inspired by approaches used for pediatric patients has led to improved remission rates in T-ALL patients; however, the prevalence of complications, including those of the central nervous system (CNS), remains higher in T-ALL patients than in patients with other ALL subtypes [2]. Therefore, therapeutic approaches based on the genomic landscape have the potential to provide novel solutions and improve the survival of patients with T-ALL.

During the transition from precursor to mature mRNA, different splicing modalities facilitate the generation of multiple unique mature mRNAs from a single gene via complex protein regulatory mechanisms. This form of regulation is referred to as alternative splicing. The differential expression of splicing factors plays an important role in cancer pathogenesis, contributing to many aspects of cancer such as oncogenic transformation, cancer progression, responses to anticancer therapy, and therapeutic resistance [3]. Genes encoding regulatory factors for precursor mRNA splicing are frequently upregulated in oncogenic contexts [4–6], and there is a prevailing view that interventions targeting spliceosome activity may offer therapeutic benefits to a broader cohort of cancer patients [7].

The field of targeted protein degradation is at the forefront of contemporary pharmacological research and development. Researchers developed indisulam (E7070), a molecular glue compound that targets splicing factor proteins. This aryl sulfonamide anticancer drug binds the U2AF-related splicing factor RNA-binding motif protein 39 (RBM39) to the CRL 4-DCAF15 E3 ubiquitin ligase, inducing rapid proteasomal degradation, abnormal RNA splicing, and cell death. RBM39 is also known as hepato-cellular carcinoma 1 (HCC1), CAPER or CAPERα [8, 9]. Studies have demonstrated that the malignant phenotypes of acute myeloid leukemia [10], breast cancer [11], colorectal cancer [12], and lung cancer [13] are maintained via RBM39. Moreover, accumulating evidence indicates that RBM39 primarily modulates the selective splicing of a diverse set of genes by facilitating exon inclusion and intron exclusion. Evidence derived from publicly available datasets robustly demonstrates that numerous lymphoma and leukemia cell line models show pronounced sensitivity to the therapeutic agent indisulam [14]. Additionally, previous research has shown that indisulam is a viable and safe therapeutic option for the treatment of AML [10].

The effects of indisulam on T-ALL have not yet been studied, and the role of its target, RBM39, in T-ALL is still unknown. Therefore, our study aimed to determine whether indisulam can also serve as an effective drug for treating T-ALL and to investigate the critical role of its target RBM39 and the key molecular mechanisms underlying its efficacy.

## Materials and methods

### Cell lines and cell culture

Human T-ALL cell lines, J.gamma1, Jurkat, MOLT4, and 6T-CEM cells, were purchased from Shanghai Zhong Qiao Xin Zhou Biotechnology Co., Ltd. The Chinese Academy of Sciences Cell Bank supplied CCRF-CEM and HUT78 cells. MV-4-11, U-937, Kasumi-1, and K-562 cells were obtained from the National Collection of Authenticated Cell Cultures in Shanghai, China. All cell lines were cultured in the recommended media. Additionally, 293FT cells (from the cell repository of the Chinese Academy of Sciences) were maintained in complete growth DMEM (BasalMedia, K211107). The authenticity of the cell lines was verified through short tandem repeat DNA profiling, and routine mycoplasma testing was conducted using the MycoAlert Kit.

### Determination of cell viability

Indisulam was solubilized in DMSO to create a 10 MM stock solution, which was then added to tissue culture media to achieve the desired final concentration. To determine the half-maximal inhibitory concentration (IC50), cells (1 × 10^4 cells/well) were treated with different concentrations of indisulam (MCE, HY-13650), ranging from 10 to 80 nM, for 72 h. Primary leukemia cells from children’s bone marrow were isolated by Ficoll-Hypaque centrifugation and seeded in 96-well plates. For cell proliferation, each group (2000 cells/well) was assessed using a CCK-8 assay (APExBIO, K1018) on days 2, 4, 6, and 8, and the absorbance was measured at 450 nm with a Bio-Rad microplate absorbance reader (Bio-Rad).

### Apoptosis assay

Apoptosis assay performed using BD Annexin V Staining Kit (BD Pharmingen, 556547) following the manufacturer’s instructions. Briefly, Cells were treated with indisulam or vehicle control for 24 or 48 h, collected and rinsed with cold PBS. Cells were resuspended in 1 × binding buffer and 5 µl of annexin V and propidium iodide (PI) were added. After gentle mixing, cells were incubated for 15 min in the dark and then analyzed by flow cytometry (Beckman, Beckman Galios™ Flow Cytometer) with 300 µL of 1 × binding buffer added to each tube.

### Western blot analysis

Whole-cell lysates were harvested using RIPA buffer (Beyotime, P0013). Cell lysates with the same concentration of protein were loaded on a pre-cast protein gel (GenScript, M42015C) and then transferred to a PVDF membrane (Merck Millipore, IPVH00010). Subsequently, the membranes were blocked at room temperature using 5% skim milk in TBST (1 × TBS, 0.1% Tween 20) and then probed with primary antibodies overnight at 4 °C. Membranes were incubated with secondary antibodies for 1 h the next day, and chemiluminescent signals were detected. The primary antibody information is detailed in Supplementary Table 1.

### Lentivirus preparation and infection

Short hairpin RNA (shRNA) sequences targeting RBM39, THOC1 and EZH2 were encoded in pLKO.1-puro lentiviral vectors supplied by IGE Biotechnology Ltd. Guangzhou, China. Envelope and packaging plasmids for lentivirus production were purchased from Addgene (pMD2.G: #12259; psPAX2: #12260; Cambridge, MA, USA). pMD2. G, psPAX2, and the transfer plasmid were cotransfected into 293FT cells using polyethyleneimine (PEI) reagent (Sigma-Aldrich, 49553-93-7) at a ratio of 4:3:1. After 6 h, the culture medium was completely replaced with fresh medium. T-ALL cells were then infected with lentivirus concentrated in the presence of 5 μg/mL Polybrene (Sigma-Aldrich) for 24 h. Stable cell lines were selected using puromycin (Sigma-Aldrich). Target sequence information is provided in Supplementary Table 1.

### RNA preparation and gel electrophoresis

Total RNA was extracted using the FastPure Cell/Tissue Total RNA Isolation Kit V2 (Vazyme, RC112-01). cDNA was prepared in a 25 μl reaction from 1000 ng of total RNA using M-MLV reverse transcriptase (Promega, M170A, M531A), RiboLock RNase inhibitor (Thermo Fisher, EO0381), random primers (Genewiz) and a dNTP mixture (Takara, 4019) along with an ABI PCR apparatus (Thermo Fisher, Applied Biosystems). A One-drop OD-1000 spectrophotometer was used to determine RNA concentration and purity. Endpoint PCR was used to assess exon skipping in EZH2 and THOC1. In total, cDNA was amplified with 2 × Taq Plus Master Mix II (Vazyme, P213) and then separated by gel electrophoresis (2% agarose). The primer information is provided in Supplementary Table 1.

### Quantitative real-time PCR (qRT‒PCR)

qRT-PCR was performed in triplicate (*n* = 3) on a LightCycler 480 Real-Time System manufactured by Roche. LightCycler® 480 SYBR Green I Master Mix (Roche, 04707516001) was used. The delta-delta Ct method was used to calculate relative expression levels between cell lines according to standard procedures. The primer information is provided in Supplementary Table 1.

### Cell cycle analysis

Cells (300,000 per well) were plated into 6-well culture plates and exposed to Indisulam for 48 h. Cells were then harvested by centrifugation at 800 rpm for 4 min at 4 °C, then fixed in 75% ethanol overnight. Samples were resuspended in PI solution (0.05 mg/ml PI, 0.1% sodium citrate, and 0.1% Triton X-100) and incubated at room temperature for 20 min. A Beckman Gallios™ Flow Cytometer (Beckman) was used to determine the distribution of cells in each cell cycle phase.

### CRISPR-mediated inactivation of DCAF15

To generate stable DCAF15-knockout cell lines using the Cas9/CRISPR system, the Cas9 gene was initially delivered into T-ALL cells through lentivirus transduction, followed by puromycin selection. Single guide RNAs (sgRNAs) targeting DCAF15 were then cloned and inserted into the Lenti-CRISPR plasmid provided by GENECHEM. Cas9-expressing cells were transduced with either sgRNA-DCAF15 or nontargeting control sgRNA (sgNC) lentiviral particles. Lentivirus preparation was conducted using a previously described method.

### Soft agar colony formation analysis

Agarose gels of 0.7% and 1.2% (SIGMA, A9045-10G) and 2 × RPMI 1640 medium (YuChun, YC-1010) supplemented with 20% FBS, penicillin, and streptomycin were prepared. A mixture of equal parts 1.2% agarose gel and medium was added to the bottom of a plate, and a mixture of equal parts 0.7% agarose gel and medium was added as a second layer. After solidification of the bottom gel layer, the top layer of gel and cells were added. The plate was incubated at 37 °C with 5% CO2, and fresh culture medium was added every 3 days. After 2–3 weeks, the cells were fixed and stained with 4% paraformaldehyde (Beyotime, P0099) and Giemsa (Beyotime, C0131), and the number of colonies was counted.

### Animal experiments

All animal protocols (CAM-SU-AP: JP-2018-1) were thoroughly reviewed and approved by the Animal Care and Use Committee of Soochow University Pediatric Clinic. Four- to five-week-old NOD scid gamma (NSG) mice (Shanghai Model Organisms Center, Inc.) were used in the experiments. The mice were randomly assigned to different groups.

*For indisulam efficacy studies in in vivo models*, an indisulam (MCE, HY-13650) stock solution was diluted in SBE-β-CD (MCE, HY-17031) to achieve a final concentration of 12.5 mg/kg. SBE-β-CD was added to saline in advance to obtain a 20% cosolvent. NSG mice were intravenously injected with 2 million luciferase-expressing J.gamma1 cells. Upon disease onset, as measured by bioluminescent imaging, the mice were intraperitoneally injected with either 12.5 mg/kg indisulam or vehicle (1% DMSO) (*n* = 6/group) once daily for 5 days on and 2 days off. Whole-body bioluminescent imaging was conducted by intraperitoneally injecting luciferin (GOLDBIO, 115144-35-9) at a concentration of 50 mg/kg, with imaging performed 5 min postinjection using a Berthold imaging apparatus every 10 days.

*For the shRNA xenograft study*, J.gamma1-expressing luciferase cells were transduced with shRNA lentiviral particles targeting RBM39. Following a 48-h incubation period, puromycin (1 μg/ml) was added for selection purposes, with the incubation period extended to 72 h. Two million cancer cells were intravenously injected into NSG mice (*n* = 6/group), and imaging was performed every 10 days. Following euthanasia via CO2 inhalation, bone, hepatic, and splenic tissue samples were collected for further experimentation, including CD45+ cell detection via flow cytometry analysis, hematoxylin and eosin staining, and immunohistochemical analyses.

### RNA sequencing and data processing

RNA isolation, library preparation, transcriptomic sequencing (Illumina NovaSeq 6000), and data cleansing were carried out by Novogene Bioinformatics Technology Co., Ltd. (Beijing, China). The 150 bp paired-end reads were aligned to the GENCODE v29 gene reference and the hg38 genome using STAR software (version 2.7.10a) with the parameters --outFilterMultimapNmax 1 --outFilterMismatchNoverReadLmax 0.04 --alignEndsType EndToEnd. Transcriptome assembly and abundance analysis were performed with StringTie software (version 2.1.7). We utilized the rMATS software (version 4.1.2) for the analysis of alternative splicing in the RNA-seq dataset, encompassing the classification of splicing events, as well as the quantitative and differential analysis of alternative splicing events.

### Protein identification and quantification by LC–MS/MS

#### Sample preparation

Protein samples were frozen with liquid nitrogen, ground, and mixed with lysis buffer. Next, the solution was sonicated on ice and centrifuged to remove debris. The supernatant was collected for subsequent analysis. For trypsin digestion, proteins were precipitated with TCA, centrifuged, washed with acetone, and dried. The peptides were redissolved in TEAB, dispersed ultrasonically, digested with trypsin overnight, reduced with dithiothreitol, alkylated with iodoacetamide, and desalted using a Strata X SPE column.

#### LC‒MS/MS analysis

Peptides were separated on a reversed-phase column using a solvent gradient and then analyzed with a timsTOF™ Pro mass spectrometer in dia-PASEF mode. The process involved a 20-min gradient, an electrospray voltage of 1.75 kV, and MS/MS scans between 300–1500 and 400–8500 m/z with a 7 m/z isolation window. Data-independent acquisition (DIA) data were analyzed with the DIA-NN search engine (v.1.8) against the Homo_sapiens_9606_SP_20231220.fasta database (20,429 entries) plus reverse decoys. The parameters included trypsin/P cleavage with ≤ 1 missed cleavage, N-terminal Met excision, carbamidomethyl on Cys as a fixed modification, and an FDR < 1%.

### Statistics and reproducibility

For statistical comparisons, we performed unpaired Student’s t tests and one-way analysis of variance (ANOVA). Survival curves were compared using the log-rank test. The data were analyzed in GraphPad Prism (v9.2.0) and R. The data shown were obtained from representative experiments with similar results. Data with statistical significance are indicated by the following symbols: * *p* < 0.05, ** *p* < 0.01, *** *p* < 0.001, and **** *p* < 0.0001.

## Results

### Indisulam exerts significant antitumor effects on T-cell acute lymphoblastic leukemia (T-ALL)

**Fig. 1:**
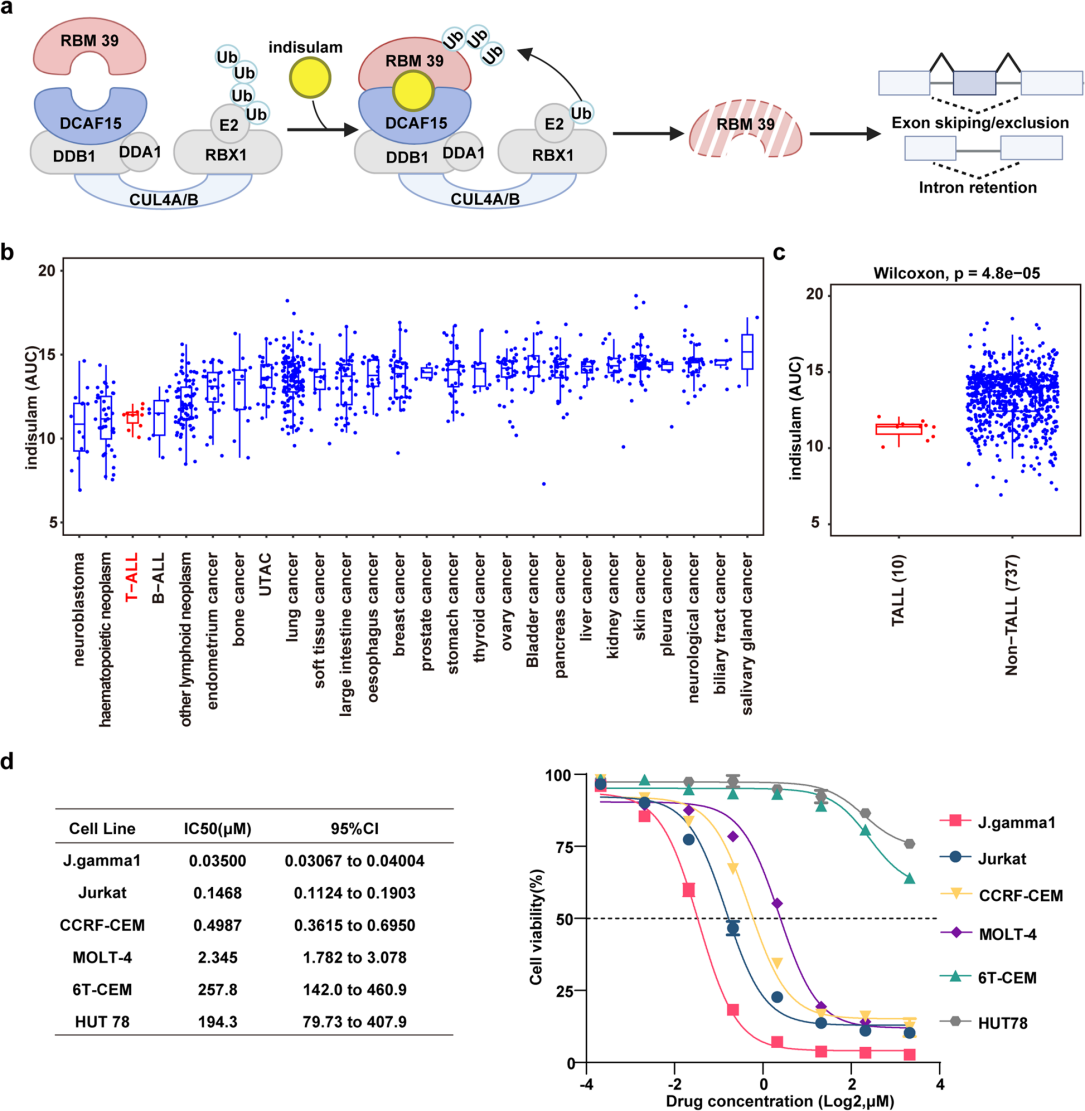
T-ALL cell lines demonstrate sensitivity to indisulam. **a** Aryl sulfonamides, exemplified by indisulam (yellow), act as molecular glue compounds, facilitating the convergence of the E3 ubiquitin ligase DCAF15 with RBM39, resulting in polyubiquitination and degradation of the protein. Depletion of RBM39 leads to aberrant splicing events. **b** Median area under the curve (AUC) across 26 tumor cell lines, with data sourced from the CTD2 network [14]; **c** The AUC values were determined for 10T-ALL cell lines and compared to those of non-T-ALL cell lines (all other cancer types, *n* = 744). **d** The drug sensitivity of the J.gamma1, Jurkat, CCRF-CEM, MOLT-4, 6T-CEM, and HUT78 cell lines following treatment with gradient concentrations of indisulam for 48 h

Indisulam, an aryl sulfonamide drug, functions as a “molecular glue” by connecting the U2AF-related splicing factor RBM39 to the CRL4-DCAF15 E3 ubiquitin ligase. The recruitment of RBM39 to CRL4-DCAF15 results in the ubiquitination of RBM39, which is then degraded by the proteasome, leading to splicing abnormalities within treated cells (Fig. 1a). Researchers from the CTD^2^ network discovered that sensitivity to indisulam was notably greater in cancer cell lines originating from hematopoietic and lymphoid (HL) cells [14]. This finding suggests that indisulam may exhibit better therapeutic effects on tumors arising from HL cell-derived tumors [12]. Consequently, our preliminary research focused on evaluating the susceptibility of tumors arising from HL cells to indisulam, utilizing data from publicly accessible databases. By assessing drug sensitivity, which was quantified through the area under the curve (AUC) values arranged in ascending order, it was observed that T-ALL cells ranked prominently in terms of sensitivity (Fig. 1b, Supplementary Table 2). Additionally, compared to non-T-ALL cell lines, T-ALL cell lines were significantly more sensitive to indisulam (Fig. 1c).

Following the initial observations, we treated six established T-ALL cell lines with indisulam in laboratory settings. After a 48-h treatment period, we performed cell viability assays and calculated the IC50 values. Remarkably, four of these cell lines exhibited notable sensitivity to the drug. Specifically, J.gamma1 and Jurkat cells were the two most sensitive cell lines (Fig. 1d). Additionally, we evaluated the sensitivity of AML cell lines, and our findings aligned with earlier research outcomes (Supplementary Figure 1) [15].

### Indisulam inhibits cell growth, disrupts the cell cycle, and induces cell apoptosis in vitro

**Fig. 2:**
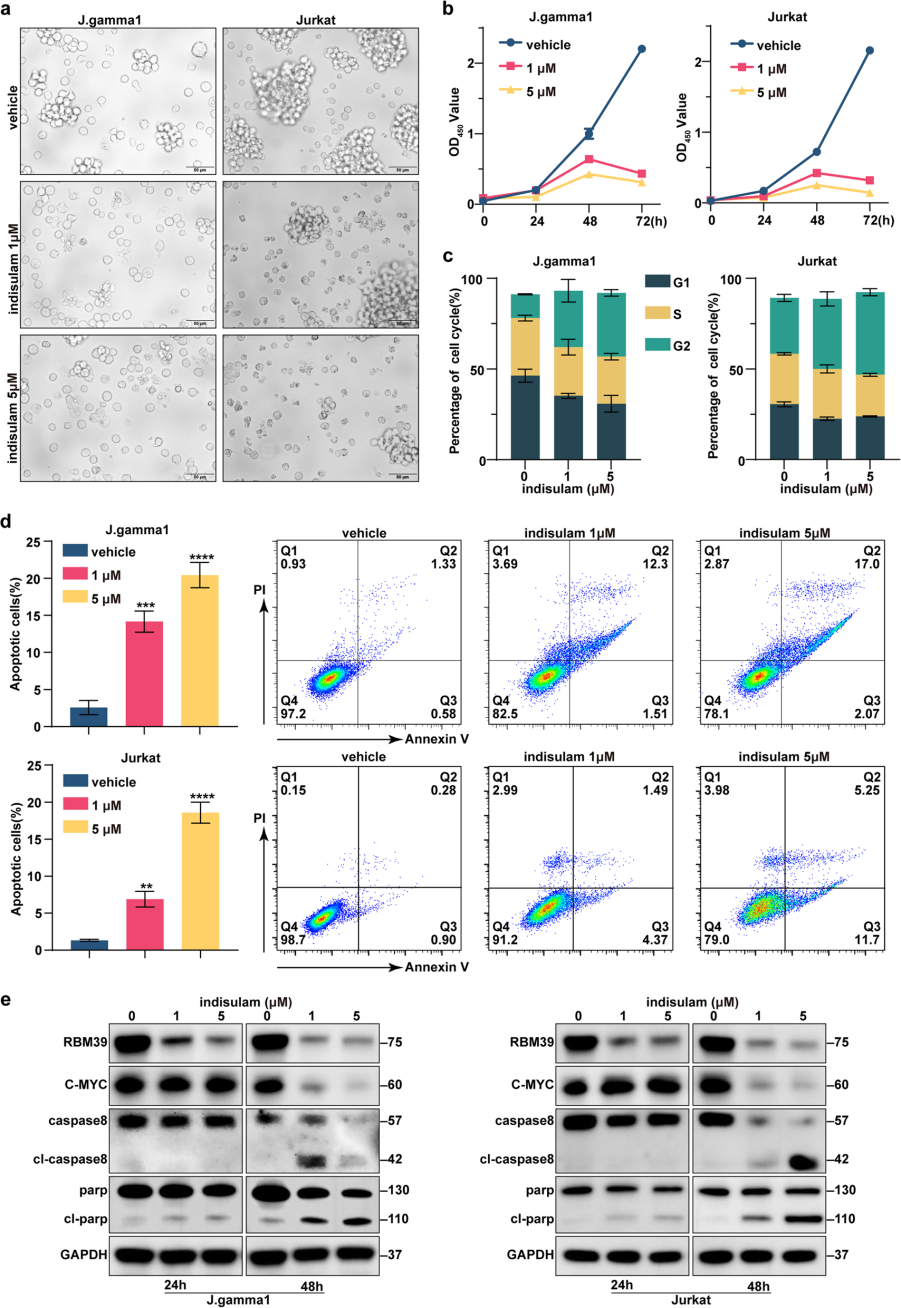
Indisulam is highly efficacious in in vitro models of T-ALL. **a** Fluorescence microscopy images of the T-ALL cell lines J.gamma1 and Jurkat after 48 h of treatment with 1 μM and 5 μM indisulam, respectively. **b** Cell proliferation curves within 72 h after treatment with 1 μM and 5 μM. **c** Cell cycle analysis was conducted on J.gamma1 and Jurkat cells labeled with PI after 48 h of treatment with DMSO or varying concentrations of indisulam. **d** Flow cytometry analysis was performed to assess apoptosis in T-ALL cells after 24 h of treatment with DMSO or various concentrations of indisulam utilizing Annexin V and PI staining. The percentage of apoptotic cells was subjected to statistical evaluation. **e** Western blot analysis was performed to validate the protein expression changes occurring in the J.gamma1 and Jurkat cell lines after treatment with indisulam for 24 and 48 h, respectively

We conducted a thorough evaluation of the efficacy of indisulam in ameliorating T-ALL by treating two T-ALL cell lines, namely, J.gamma1 and Jurkat cells, with indisulam at concentrations of 1 μmol and 5 μmol, respectively. Within 48 h post-indisulam administration, fluorescence microscopy revealed that a significant proportion of the T-ALL cells died (Fig. 2a). Continuous viability assays conducted 72 h after drug administration demonstrated a marked decrease in the viability of all tested cell lines in a time-dependent manner (Fig. 2b). Western blot analysis confirmed a dose-dependent decrease in the proliferation marker protein c-Myc following drug exposure (Fig. 2e). Additionally, PI staining indicated that indisulam induced cell cycle arrest, significantly increasing the proportion of T-ALL cells in the G2 phase while reducing the proportion of cells in the G1 phase (Fig. 2c, Supplementary Figure 2a). This finding demonstrated that indisulam inhibits cell proliferation by regulating the cell cycle process. Exposure to lethal doses of indisulam for 48 h significantly increased the percentage of apoptotic T-ALL cells (Fig. 2d). Notably, although a significant reduction in the target protein RBM39 was observed after 24 h of drug treatment (Fig. 2e), apparent cell apoptosis was not evident (Supplementary Figure 2b). Further evaluation of apoptosis-related proteins, such as cleaved-PARP and cleaved-caspase8, through Western blot analysis revealed a notable increase in all treated cells, validating the results of previous experiments (Fig. 2e). In summary, the findings show that indisulam exhibits profound antitumor activity in T-ALL in vitro models.

### Indisulam has a significant inhibitory effect on T-ALL and has fewer toxic side effects in vivo

**Fig. 3:**
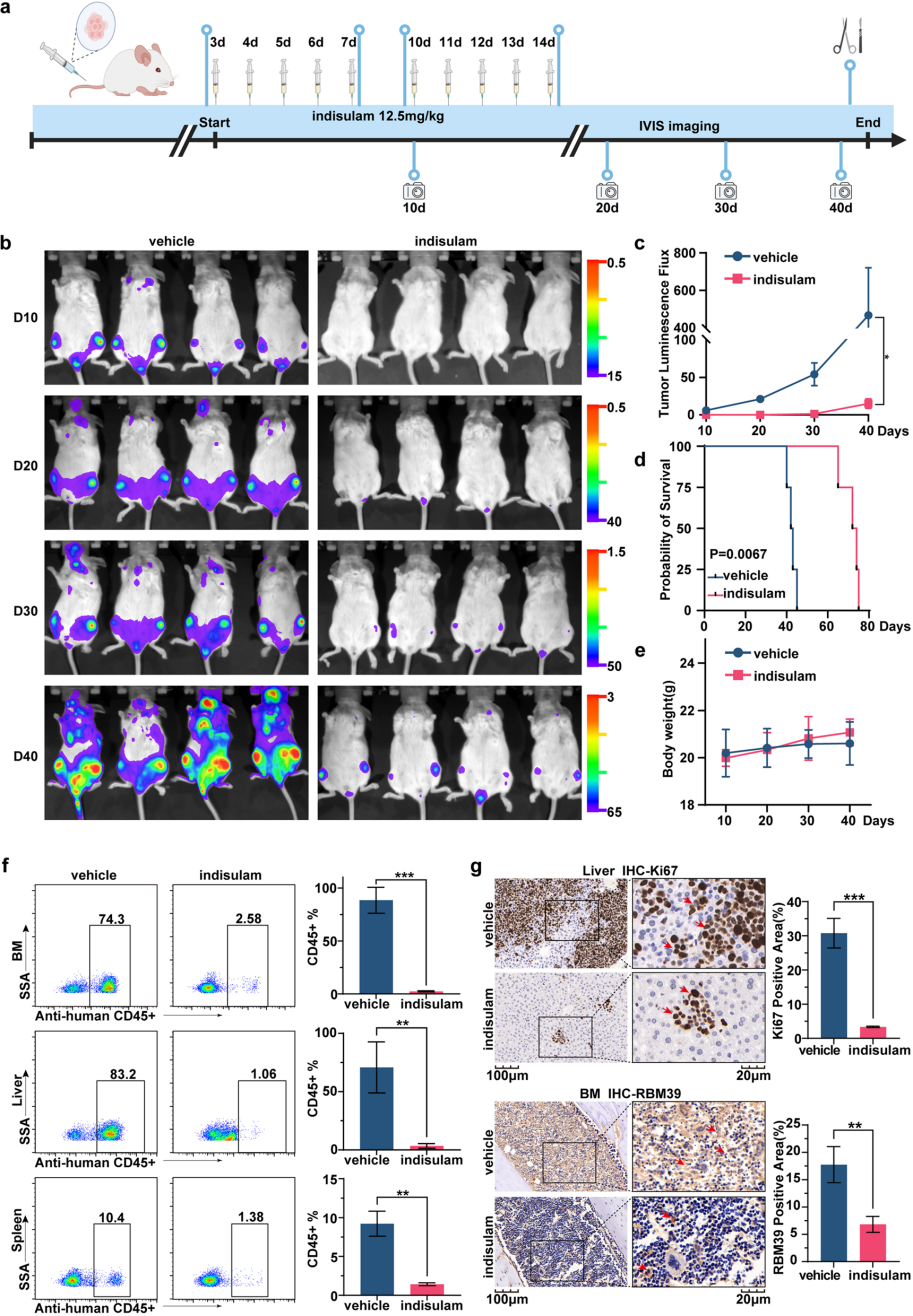
Indisulam treatment elicits a significant response in T-ALL in vivo models. **a** Schematic diagram of the in vivo experiment. **b** Imaging of the mice was performed every ten days. **c** Comparison of fluorescence values between the two groups of mice, which served as an indicator of the extent of leukemia burden in the mice. **d** Kaplan-Meier curves illustrating the survival rates of NSG mice following indisulam treatment. **e** Body weight changes in the two preclinical leukemia mouse models. **f** After the incubation of mouse liver, spleen and bone marrow cells with an anti-human CD45 antibody, the percentage of positive cells was quantitatively assessed and subjected to statistical analysis. **g** Representative images of mouse bone marrow and liver immunohistochemical staining. Immunohistochemistry was used to determine the RBM39-positive regions and Ki67-positive regions

To further assess the therapeutic efficacy of indisulam, a preclinical model of T-ALL was established by utilizing J.gamma1 cells. The experimental protocol is delineated in Fig. 3a. Indisulam was administered intraperitoneally at a dose of 12.5 mg/kg [16], while the control group received the same volume of a vehicle solution through the same route. The treatment protocol included five consecutive days of administration followed by a two-day break, and this cycle was repeated twice.

Every ten days after the start of treatment, in vivo imaging was performed using the NightOWL In Vivo Imaging System to monitor the leukemia burden based on fluorescence intensity. Compared with the control group, the indisulam-treated group displayed a significant decrease in fluorescence intensity at four distinct time points posttreatment, demonstrating a substantial suppression of J.gamma1 cell proliferation in vivo (Fig. 3b-c). A comparison of the survival times of the two groups revealed that indisulam can prolong the lifespan of mice. (73 ± 1.7 vs. 42.5 ± 0.6 days, *P* = 0.0067) (Fig. 3d). Importantly, no notable differences in body weight were observed between the two groups, indicating the absence of significant side effects from indisulam treatment (Fig. 3e). Flow cytometric analysis of the liver, spleen, and bone marrow from the mice stained with a human CD45+ antibody revealed a significant decrease in the percentage of cells that were positive for J.gamma1, underscoring the efficacy of the drug in these tissues (Fig. 3f). Moreover, HE staining and immunohistochemical analyses of the mouse bone, liver, and spleen tissues revealed a noticeable reduction in tumor cell infiltration (Supplementary Figure 3) and decreased expression of Ki67 and RBM39 (Fig. 3g). These results collectively highlight the durable antitumor efficacy of indisulam in an in vivo T-ALL model, confirming its potential as an effective therapeutic agent.

### Knocking down the indisulam target RBM39 has anticancer effects on T-ALL in vitro

**Fig. 4:**
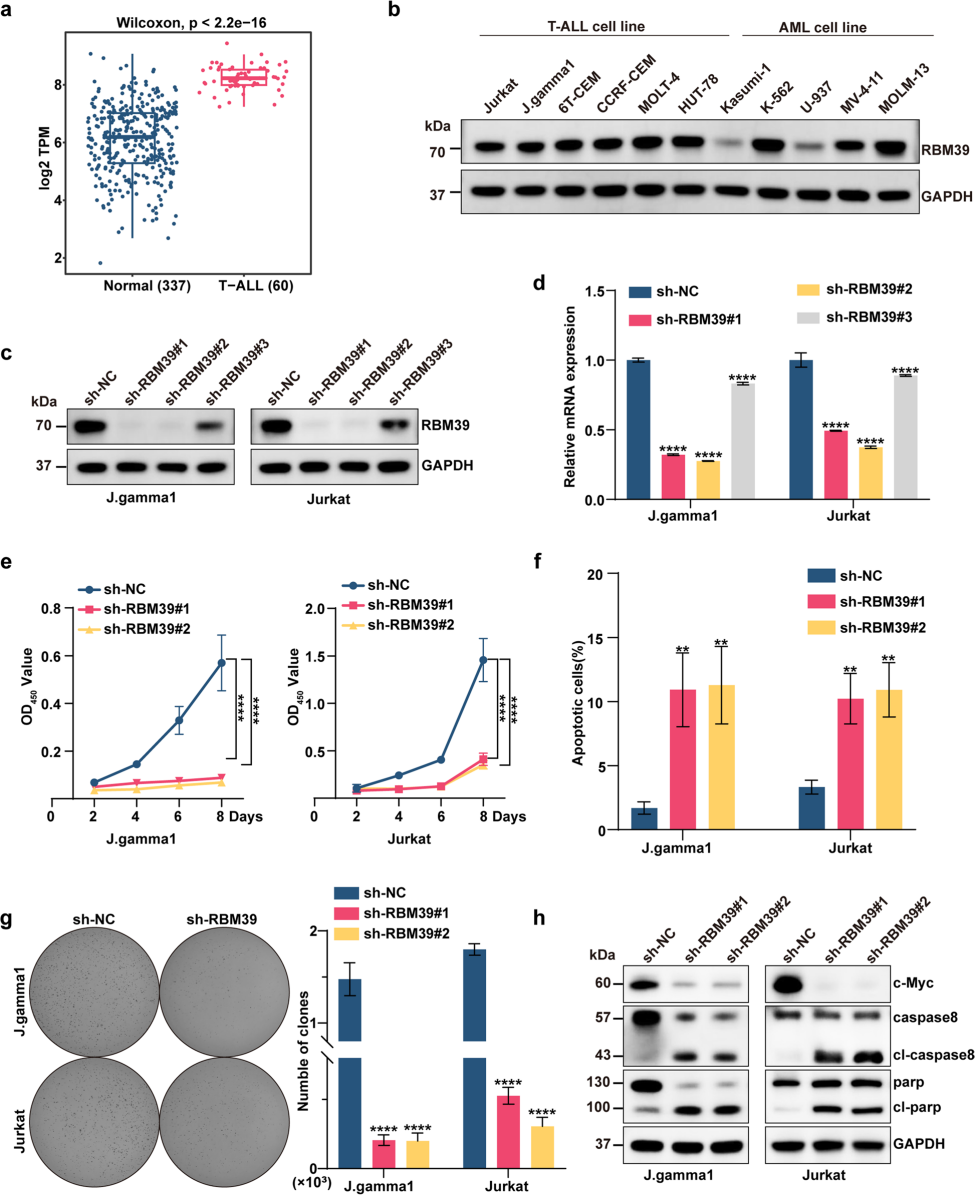
Indisulam exerts its anticancer effects by targeting RBM39. **a** Transcriptomic data revealed differences in RBM39 expression between individuals diagnosed with T-ALL and healthy subjects. The T-ALL data is from GSE110637 and the normal data is from whole blood in GTEx. **b** The protein expression levels of RBM39 in different cell lines. The first six were T-ALL cell lines, while the last five were AML cell lines. **c** The knockdown efficiency of RBM39 was evaluated in the J.gamma1 cell line and Jurkat cell line by Western blotting.table. **d** The knockdown efficiency of RBM39 was evaluated in the J. gamma1 cell line and Jurkat cell line by qRT‒PCR. **e** Moreover, RBM39 knockdown significantly inhibited the proliferation of J. gamma1 and Jurkat cells. **f** Flow cytometry analysis indicated that RBM39 depletion led to a notable increase in apoptosis. **g** The knockdown of RBM39 affects cell colony formation and proliferation. **h** Western blot analysis validated the protein alterations occurring in the J.gamma1 and Jurkat cell lines following shRNA-mediated knockdown

Our aim was to ascertain whether the anticancer effects of indisulam on T-ALL are mediated via RBM39. In comparison to healthy tissue samples, RBM39 mRNA is significantly upregulated in 60T-ALL samples (Fig. 4a, Supplementary Table 3). This elevated expression of RBM39 was further validated through Western blotting in various T-ALL cell lines, including Jurkat, J.gamma1, 6T-CEM, CCRF-CEM, MOLT-4, and HUT-78 (Fig. 4b). To further explore this relationship, we knocked down RBM39 in two T-ALL cell lines (J.gamma1 and Jurkat) via shRNA constructs and confirmed the efficacy of the knockdown at both the mRNA and protein levels (Fig. 4c-d). Upon RBM39 knockdown, we noted a significant decrease in the proliferation rate of T-ALL cells (*P* < 0.001) (Fig. 4e), accompanied by a corresponding downregulation of the proliferation-related protein c-Myc (Fig. 4h). This effect was accompanied by a pronounced suppression of colony formation and proliferation (Fig. 4g). Flow cytometric analysis indicated an increase in apoptosis among T-ALL cell lines after RBM39 suppression (Fig. 4f, Supplementary Figure 4). Moreover, the decrease in RBM39 levels led to an increase in the levels of the apoptosis-inducing proteins cleaved-PARP and cleaved-caspase8 (Fig. 4h). Collectively, these findings underscore the critical role of RBM39 in the survival of T-ALL cells, demonstrating that the anticancer effects of indisulam are mediated via RBM39.

### Knocking down RBM39 markedly impedes T-ALL progression in vivo

**Fig. 5:**
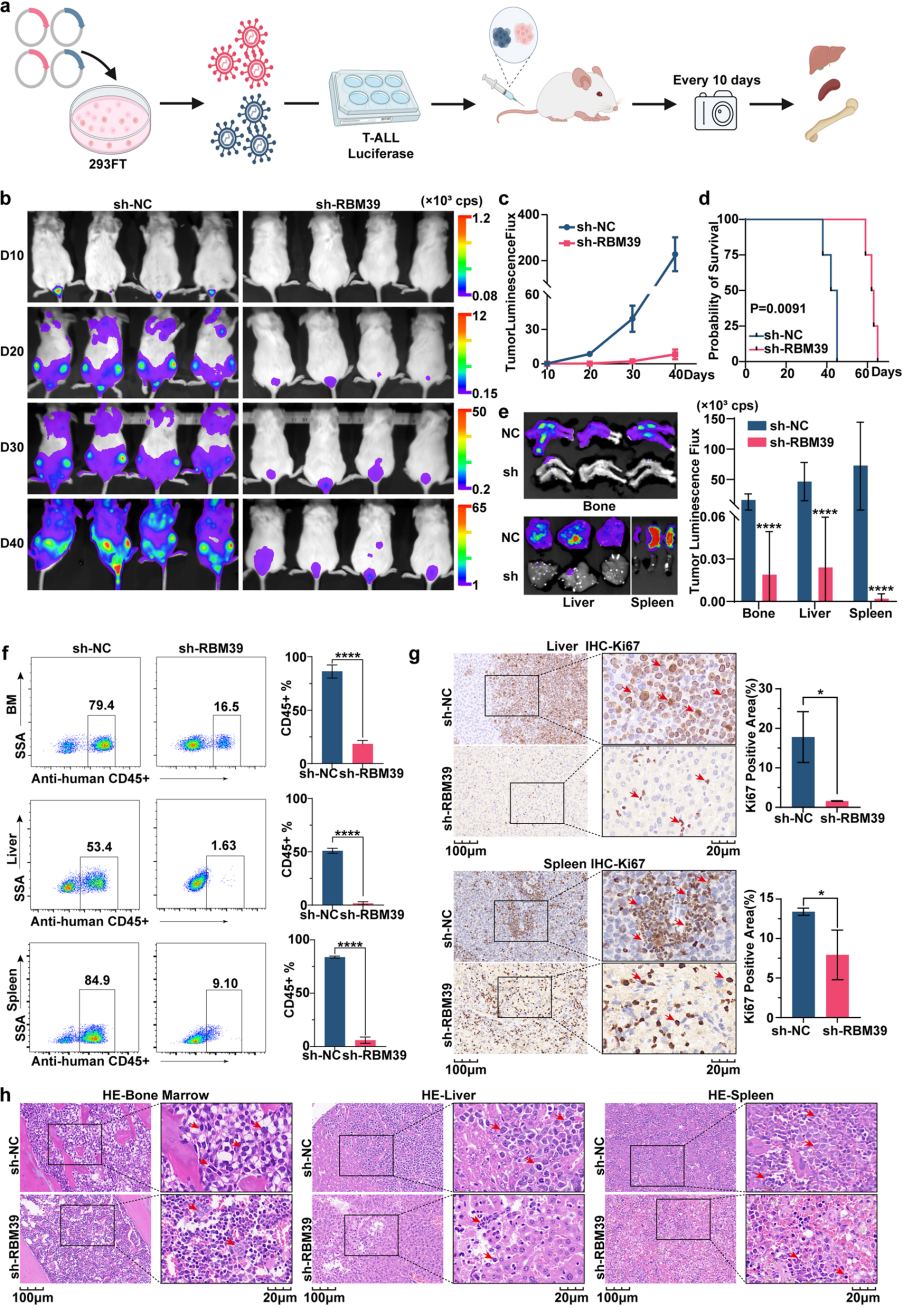
In vivo experiments validated the role of RBM39 in T-ALL. **a** Conceptual representation of a T-ALL cell xenograft model featuring the injection of RBM39-depleted cells with luciferase reporter activity. **b** Fluorescence images of mice captured at four distinct time points. **c** Statistical analysis of in vivo fluorescence intensity in both mouse groups. **d** Kaplan‒Meier curves displaying the survival duration of the mice. **e** Fluorescence images of mouse liver, spleen, and bone, followed by quantitative analysis of fluorescence intensity. **f** Flow cytometry analysis revealed the proportion of human CD45-positive cells in mouse tissues. **g** Immunohistochemistry was used to determine the Ki67-positive regions. **h** Hematoxylin and eosin staining assessment of bone marrow, liver, and spleen in the two groups

The suppression of leukemia progression through RBM39 knockdown has been demonstrated, leading to the initiation of in-depth in vivo investigations. We knocked down RBM39 in J.gamma1 cells and transfected them with a luciferase reporter before transplantation into NSG mice (Fig. 5a). The in vivo fluorescence values represented leukemia burden. Compared with control treatment, RBM39 knockdown resulted in a significant reduction in J.gamma1 cell expansion in vivo at four time points (Fig. 5b). The line chart of the tumor fluorescence data illustrates that the fluorescence values of the RBM39 knockdown group was markedly lower than those of the control group (Fig. 5c). Survival analysis between the two groups indicated a pronounced increase in the lifespan of mice in the RBM39-suppressed group (Fig. 5d). Examination of fluorescence in the liver, spleen, and bone marrow of mice implanted with RBM39-suppressed cells revealed a significant decrease in leukemia burden compared to that in control mice (Fig. 5e). Additionally, there was a significant decrease in the number of leukemia cells in the RBM39 group compared to the control group, which was indirectly indicated by the proportion of anti-human CD45+ cells (Fig. 5f). Immunohistochemical assessments also revealed a decrease in Ki67-positive proliferating cells in the RBM39-suppressed group (Fig. 5g, Supplementary Figure 5). Moreover, detailed histological analyses of liver and spleen samples confirmed reduced infiltration of RBM39-suppressed J.gamma1 cells in these organs (Fig. 5h). In conclusion, these findings confirm that RBM39 deletion markedly impedes leukemia progression in vivo, highlighting its potential as a therapeutic target in T-ALL management.

### Mis-splicing of mRNA is the mechanism by which targeting RBM39 through indisulam exerts anti-cancer effects in T-cell acute leukemia

**Fig. 6:**
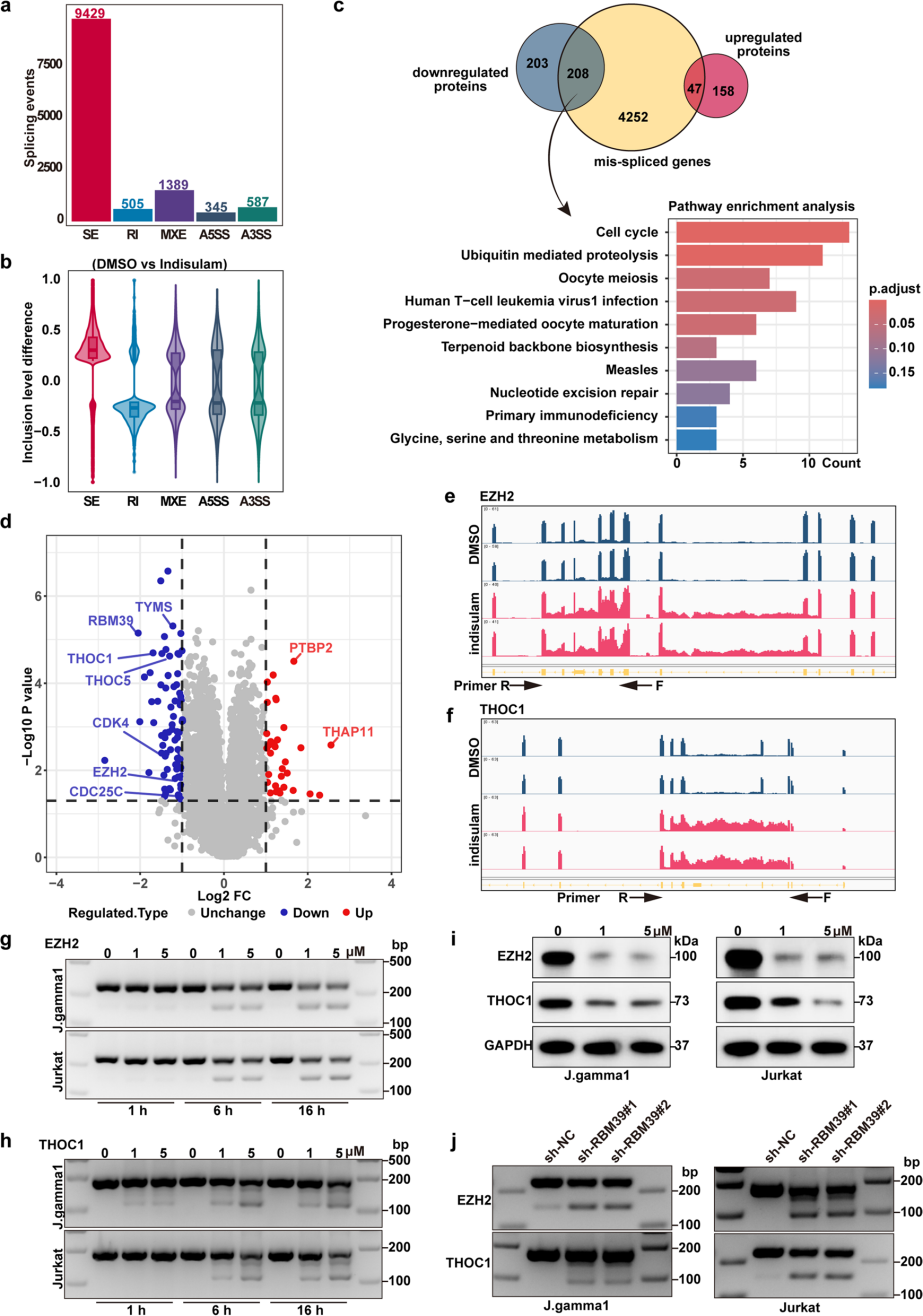
Indisulam mediates the elimination of RBM39, leading to widespread downstream mis-splicing events. **a** RNA-seq analysis of the types and numbers of alternative splicing events that occur after indisulam treatment. **b** RNA-seq analysis of the propensity for various types of events following indisulam treatment. **c** Overlap of splicing events (exon skipping and intron retention) with up- or downregulated proteins after 16 h of indisulam treatment. Gene Ontology analysis of mis-spliced downregulated proteins and pathway analysis. **d** Proteomic analysis, visualized with a volcano plot, highlighted upregulated (red) and downregulated (blue) proteins in J.gamma1 cells compared with those in control cells and indisulam-treated cells. **e** The number of RNA reads for EZH2 post-indisulam treatment was counted. The plot was generated with Integrative Genomics Viewer (IGV). The green box indicates the regions where mis-splicing events occurred. The arrow indicates the plot of custom primers used to detect exon skipping. **f** Count of RNA reads for THOC1 post-indisulam treatment. The plot was generated with IGV. The green box indicates the regions where mis-splicing events occurred. The arrow indicates the plot of custom primers used to detect exon skipping. **g** PCR analysis of EZH2 in J. gamma1 and Jurkat cells treated with indisulam. **h** PCR analysis of THOC1 in J.gamma1 and Jurkat cells treated with indisulam. **i** Western blot analysis of EZH2 and THOC1 after indisulam treatment. **j** PCR analysis of EZH2 and THOC1 in RBM39-knockdown cells following indisulam treatment

According to previous research, high doses of indisulam can directly induce RNA splicing events within 16 h, bypassing signaling from indirect pathways [17]. To understand the impact of aberrant alternative splicing on protein levels, we performed transcriptomic and proteomic analyses of the J.gamma1 cell line after indisulam treatment at a later time point (5 µM, 16 h). By utilizing rMATS for analysis, distinct patterns of exon and intron mis-splicing events were observed. Among all splicing events, 9429 instances of exon skipping were observed, representing the largest proportion of mis-splicing events (Fig. 6a, Supplementary Tables 4–8). Exon skipping events were the most common mis-splicing event in exons, while intron retention events were the most common in introns (Fig. 6b). When compared with events of alternative splicing, we observed that downregulated proteins exhibited considerable overlap with transcripts exhibiting alternative splicing (208 of 411 downregulated proteins exhibited alternative splicing, representing 51% of the total, while 47 of 205 upregulated proteins demonstrated alternative splicing, representing 23% of the total). In order to gain further insight into the pathways affected by this mechanism, the 208 target genes underwent enrichment analysis. This analysis revealed that pathways linked to both the cell cycle and ubiquitin-mediated proteolysis were particularly impacted by the mechanism in question (Fig. 6c, Supplementary Tables 9–10). Through differential protein analysis between the control and drug-treated cohorts, 616 differentially expressed genes were identified (comprising 205 upregulated and 411 downregulated genes) (Fig. 6d, Supplementary Table 11). In T-ALL cells treated with indisulam, a significant number of pre-RNAs, such as those of EZH2, THOC1, TYMS, CDC25C, and THOC5, were observed to undergo exon skipping and intron retention events (Supplementary Figure 6). The exon skipping observed in EZH2 is consistent with the results of two previous studies (Fig. 6e) [12, 17]. Moreover, we are the first to discover a splicing disorder in THOC1 (Fig. 6f). PCR assays were employed to confirm the dose- and time-dependent exon skipping (Fig. 6g-h). Subsequently, Western blot analysis revealed the loss of EZH2 and THOC1 signals in the J.gamma1 and Jurkat cell lines after 48 h of drug treatment (Fig. 6i). To confirm that these events are associated with the loss of RBM39 rather than lineage specificity, RNA was extracted from RBM39-knockdown T-ALL cells, and PCR was utilized to confirm the occurrence of similar mis-splicing events (Fig. 6j). In summary, these findings illuminate that indisulam exerts its antitumor effects through the targeted elimination of RBM39, leading to extensive mis-splicing of key downstream genes.

### THOC1 is a critical downstream effector of indisulam and RBM39

**Fig. 7:**
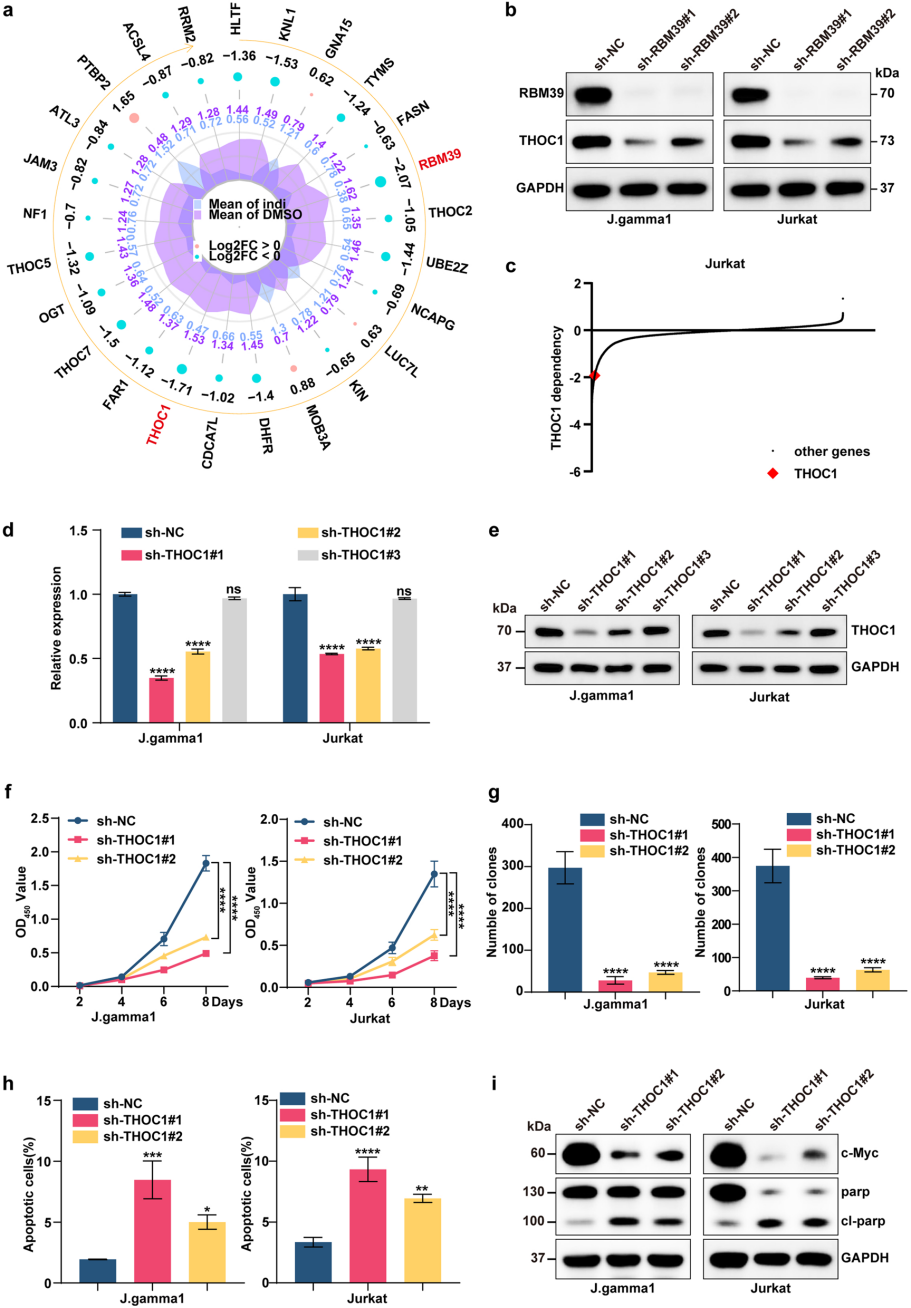
THOC1 was identified as a critical downstream target within the pharmacological mechanism of indisulam. **a** The radar chart illustrates the extent of protein expression differences. From the inside out, the first ring arranges differential proteins clockwise according to their P values from smallest to largest. The second ring displays the fold change of differential proteins between the experimental group and the control group after Log2 transformation, with pink indicating upregulation, light blue indicating downregulation, and larger dots representing greater fold changes. The fourth ring shows the average quantitative values of both groups. **b** Western blot analysis of EZH2 and THOC1 in RBM39-knockdown cells following indisulam treatment. **c** CRISPR screening data from CCLE CRISPRi revealed the dependence of T-ALL Jurkat cell survival on THOC1. **d** The knockdown efficiency of THOC1 was evaluated in the J. gamma1 cell line and Jurkat cell line by qRT‒PCR. **e** The knockdown efficiency of THOC1 was evaluated in the J. gamma1 cell line and Jurkat cell line by Western blotting. **f** Knockdown of THOC1 significantly inhibited the proliferation of J. gamma-1-expressing and Jurkat cells. **g** The knockdown of THOC1 affected cell colony formation and proliferation. **h** Flow cytometry analysis indicated that the depletion of THOC1 led to a notable increase in apoptosis. **i** Western blot analysis validated the protein alterations occurring in the J.gamma1 and Jurkat cell lines following shRNA-mediated knockdown

By employing a radar chart for the visualization of protein expression variations, after RBM39, which exhibited the most pronounced disparity, THOC1 emerged as the protein with the greatest expression changes (Fig. 7a). Additionally, Western blot analysis revealed the absence of THOC1 signals following RBM39 depletion (Fig. 7b). Additionally, CRISPR screening data from CCLE CRISPRi revealed the dependence of T-ALL Jurkat cell survival on THOC1 (Fig. 7c, Supplementary Table 12). To delineate the biological function of THOC1 within the context of T-ALL, we engineered lentiviral shRNA vectors incorporating three unique sequences targeting THOC1. Notably, compared with the negative control (sh-NC), sh-THOC#1 and sh-THOC#2 effectively suppressed THOC1 expression in the T-ALL cell lines J.gamma1 and Jurkat (Fig. 7d-e). Suppressing THOC1 expression notably hindered colony formation and proliferation in T-ALL cells, concurrently increasing apoptosis rates (Fig. 7f-g, Supplementary Figure 7a-b). Additionally, our Western blot analyses revealed to the upregulation of cleaved PARP and cleaved caspase8 (Fig. 7i). This evidence supports the hypothesis that THOC1 acts as a critical downstream target in the mechanism of action of indisulam in T-ALL.

### DCAF15 plays a crucial role in the degradation of RBM39

**Fig. 8:**
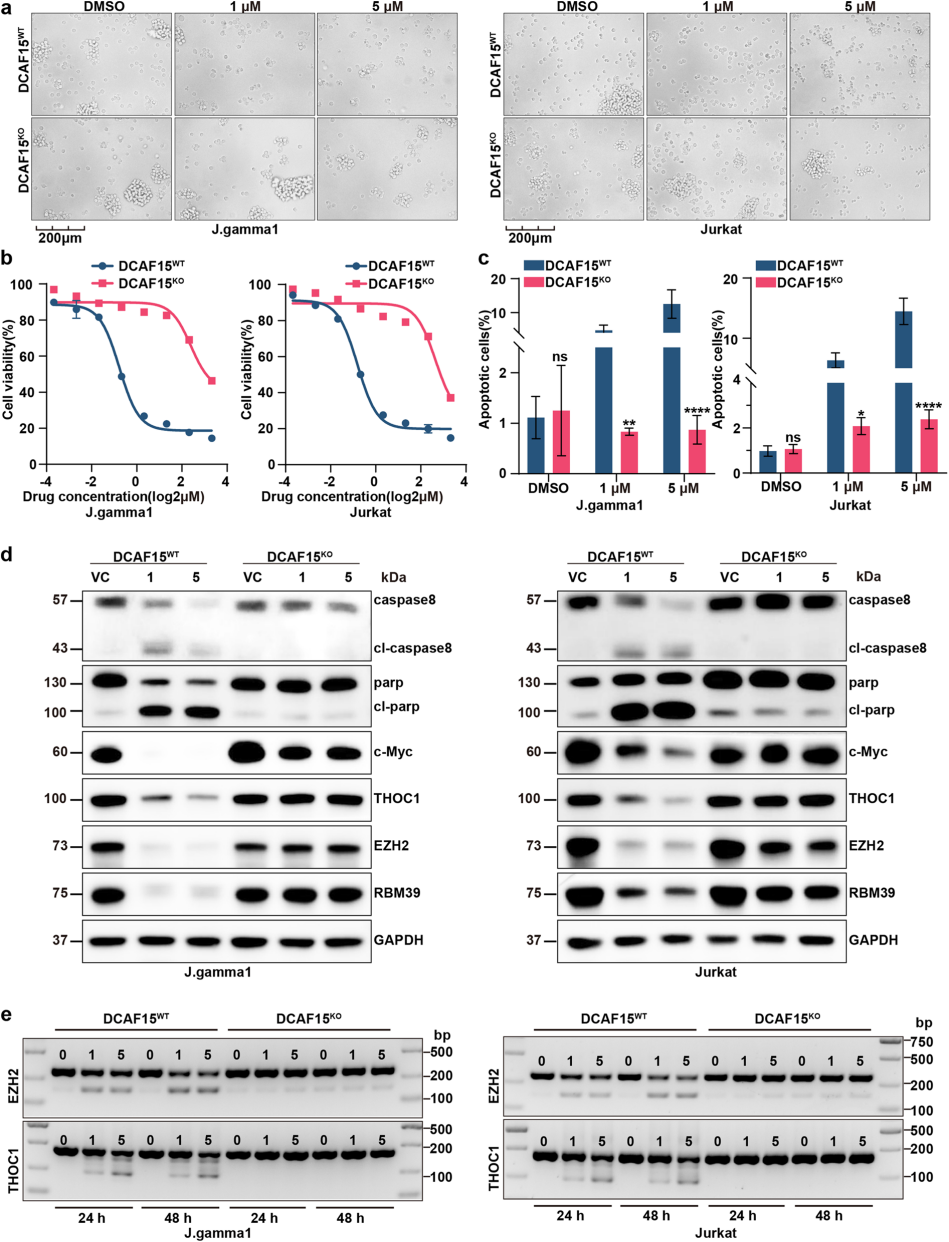
Essential role of DCAF15 in the mode of action of indisulam. **a** Representative images of DCAF15^WT^ or DCAF15^KO^ cells treated with vehicle or with 1 μm or 5 μm indisulam. **b** Cell growth was assessed after 72 h of treatment with indisulam using a CCK-8 assay. **c** The proportions of apoptotic DCAF15^KO^ and DCAF15^WT^ cells after drug treatment were measured by flow cytometry. **d** Western blot analysis confirmed the protein alterations caused by DCAF15 deficiency. **e** PCR analysis of EZH2 and THOC1 exon skipping in DCAF15^KO^ and DCAF15^WT^ cells treated with indisulam

It has long been recognized that the expression of DCAF15 is a prerequisite for the action of sulfonamide-based drugs, including indisulam. To highlight the critical function of DCAF15 in the mechanism of action of indisulam against T-ALL, we employed CRISPR-Cas9 technology to generate DCAF15-null variants in both the J. gamma1 and Jurkat cell models. Under microscopic observation, we noted that the knockout of DCAF15 rendered indisulam ineffective in inducing cytotoxicity in T-ALL cells (Fig. 8a). Remarkably, we did not observe the loss of RBM39 protein in cells lacking DCAF15 (Fig. 8d). Furthermore, in DCAF15-knockout clones, the inhibitory effect on proliferation and the promotive effect on apoptosis exerted by indisulam were restored (Fig. 8b-c, Supplementary Figure 8). Moreover, the mis-splicing events of EZH2 and THOC1 induced by drug action were conspicuously absent in cells with DCAF15 deficiency (Fig. 8e). Consistently, protein loss resulting from exon skipping was also not observed (Fig. 8d). Compared to the other four cell lines, the 6T-CEM and HUT78 cell lines were found to be less sensitive to indisulam. Despite RBM39 degradation after indisulam treatment, we did not observe the depletion of downstream THOC1 (Supplementary Figure 9a). Due to the lack of specificity in DCAF15 antibodies, it has been challenging to investigate whether the sensitivity to indisulam is associated with the protein expression levels of DCAF15 [18]. Furthermore, PCR analysis indicated that this process was independent of DCAF15 mRNA expression (Supplementary Figure 9b). Therefore, we speculate that the resistance mechanism in HUT78 and 6T-CEM cells is due to the aberrant splicing of effector genes. In summary, our findings underscore the indispensable role of DCAF15 in the mechanism of action of aromatic sulfonamide drugs in T-ALL and its crucial contribution to subsequent downstream consequences.

### Indisulam has potent antitumor effects on primary T-cell acute leukemia cells

**Fig. 9:**
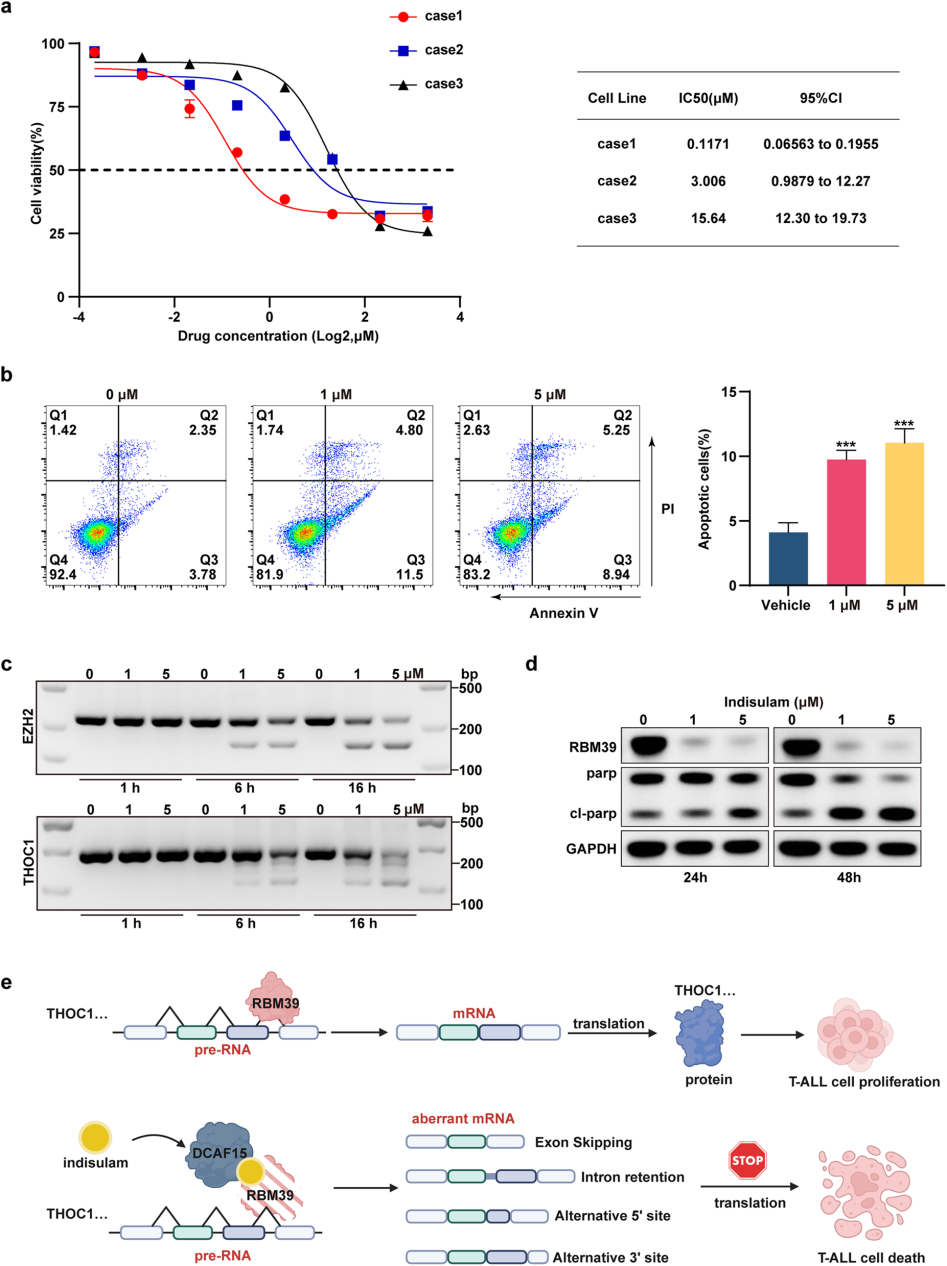
Indisulam is cytotoxic to primary T-ALL cells. **a** The drug sensitivity of primary cells treated with gradient concentrations of indisulam for 48 h was determined. **b** Following a 48-h incubation with DMSO or various concentrations of indisulam, apoptotic activity in primary cells was quantified by flow cytometry. The percentage of apoptotic primary cells increased significantly in the indisulam-treated group. **c** PCR analysis of EZH2 and THOC1 in primary cells treated with indisulam. **d** Western blot analysis revealed that indisulam induced the degradation of the RBM39 protein, decreased the level of the c-Myc protein, and increased the level of PARP in these primary T-ALL cells. **e** Pattern diagram. Indisulam recruits DCAF15 to bind to RBM39, leading to degradation of the RBM39 protein, which affects RNA alternative splicing and causes aberrant THOC1 splicing and other translational abnormalities. Deletion of THOC1 slows proliferation, increases apoptosis and disrupts the cell cycle in T-ALL cells, ultimately leading to T-ALL cell death

Three peripheral blood samples from diagnosed pediatric T-ALL patient 3 were utilized to assess the sensitivity of primary pediatric T-ALL cells to indisulam treatment. Consistent with prior observations in cell lines, cell death was noted 72 h posttreatment (Fig. 9a), as was the absence of the target protein RBM39 according to the Western blot analysis (Fig. 9d). Apoptosis increased 48 h after drug administration (Fig. 9b). Furthermore, splicing events for EZH2 and THOC1 in primary cells posttreatment were confirmed, consistent with the cell line outcomes (Fig. 9c). These findings reinforce the therapeutic potential of indisulam and lay the groundwork for its future clinical translation. Overall, indisulam recruits DCAF15 to bind to RBM39, leading to degradation of the RBM39 protein, which affects RNA alternative splicing and causes aberrant THOC1 splicing and other translational abnormalities. Deletion of THOC1 slows proliferation, increases apoptosis and disrupts the cell cycle in T-ALL cells, ultimately leading to T-ALL cell death (Fig. 9e).

## Discussion

T-ALL is a severe malignancy characterized by a high incidence of complications and a poor prognosis [19]. RNA alternative splicing plays a pivotal role in regulating cellular proliferation, survival, and differentiation, underpinning the integrity and functionality of cells [20]. Comprehensive CRISPR/Cas9 domain-focused screening targeting the RNA binding domains of 490 classical RBPs revealed a significantly upregulated network of physically interacting RBPs in AML, which is crucial for maintaining RNA splicing processes and AML cell survival [10]. Research indicates that out of the 58 and 45 RBPs analyzed in HepG2 and K562 cells, respectively, approximately 60% strongly correlated with chromatin. This suggests that RBPs not only are involved in posttranscriptional modifications but also directly participate in transcriptional regulation [21].

Using rMATS, a method for quantitatively analyzing differential splicing events, to assess cluster splicing phenomena between peripheral CD3+ T cells and T-ALL cells, it was observed that exon skipping events are markedly more prevalent in T-ALL cells than in normal T cells [22]. In Moreover, the splicing regulatory gene SHQ1 has been shown to facilitate the survival of T-ALL tumor cells via the NOTCH1-SHQ1-MYC pathway, in addition to its involvement in regulating aerobic glycolysis [23]. A preclinical study on T-ALL indicated that the splicing factor SF3B1 inhibitor E7107, when used in combination with chemotherapy drugs, exhibits a promising synergistic antitumor effect [24]. Consequently, cancer treatment strategies focusing on the targeting of alternative splicing genes are garnering increased attention among the research community.

Our research identified indisulam as an effective and safe targeted therapy for T-ALL. Indisulam, also known as E7070, was developed by Eisai Co., Ltd. Our research showed that it can induce G2 phase arrest in T-ALL cells, promoting apoptosis and inhibiting tumor cell proliferation. In vivo experiments demonstrated that drug treatment significantly extended the survival period of T-ALL-bearing mice. There is a marked reduction in the infiltration of leukemia cells into tissues such as the bone marrow and spleen. Moreover, throughout the treatment period, there were no significant changes in the body weight of the mice, nor were there any observed drug-related tissue damage. Similar to our findings, studies on several solid tumors have also reported the effective antitumor activity of indisulam [25–27]. Researchers has investigated the cytotoxic effects of indisulam on neuroblastoma, which achieves antitumor activity through the regulation of selective splicing of enzymes involved in glutamine metabolism by controlling JMJD6 [16, 28, 29]. The application of isotope tracing technology demonstrated that indisulam causes metabolic disorders and mitochondrial dysfunction in the neuroblastoma Kelly cell line [17]. Research by Ting Han, Maria Goralski, et al. revealed that indisulam exerts it effects by binding to DCAF15 and proposed that indisulam might have broader application prospects in hematologic tumors with high expression of DCAF15 [12]. A subsequent clinical trial revealed that the response rate for patients with heavily pretreated AML treated with indirubin combined with idarubicin and cytarabine was 35% [30]. Therefore, our research provides a potential basis for the application of indisulam in treating T-ALL.

Our study also revealed that RBM39 is a key gene in T-ALL cell survival. By constructing in vivo and in vitro models with RBM39 interference, we found that RBM39 is crucial for the proliferation and survival of T-ALL tumor cells. The expression of RBM39 in T-ALL tissues was significantly greater than that in normal tissues. Whether RBM39 has prognostic value in T-ALL is currently unclear. Currently, there are few functional studies on RBM39. Recent research has shown that RBM39 mediates the increase in asparagine synthesis in liver cancer, leading to enhanced arginine uptake and the formation of a positive feedback loop to maintain high levels of arginine, thus promoting carcinogenic metabolism [31]. Our experimental data also revealed that RBM39 may regulate the metabolism of T-ALL cells. Bioinformatics analysis revealed that the absence of RBM39 leads to significant changes in the glycine, serine, and threonine metabolism pathways, which is a question worth investigating in the future.

Another finding of our study is that indisulam induces widespread dysregulation of alternative splicing, disrupting the expression of proteins. By analyzing genes that undergo alternative splicing via RNA-seq and exhibit a decrease in protein levels after treatment with indisulam, we identified 208 candidate genes, including THOC1, EZH2, CHEK1, CDK4, and TRIM27. Pathway analysis revealed that these genes mainly affect the cell cycle and ubiquitin-mediated proteolysis pathways. This finding is similar to previous findings in neuroblastoma [17].

Among the numerous downstream effector changes induced by indisulam, our database search revealed that the survival of the Jurkat cell line is highly dependent on the THOC1 gene. Moreover, among the top 25 proteins according to *p* value, THOC1 ranks second only to the target protein RBM39. Therefore, we verified that THOC1 is an important downstream gene. Our experimental results showed that after THOC1 is disrupted, the growth of T-ALL tumor cells significantly slows, apoptosis increases, and the cleavage of the apoptotic molecule PARP significantly increases. Previous research has shown that the ribonucleoprotein encoded by THOC1 plays a crucial role in regulating the balance of myeloid progenitor cell proliferation and apoptosis in adult mice [32], and THOC1 deficiency also inhibits the proliferation of hepatocellular carcinoma cells [33].

DCAF15 is the key gene determining the degradation of RBM39 by indisulam, a point that has been confirmed by our research and other scientific work. After knocking out DCAF15, tumor cells become resistant to indisulam, and even 5 µM indisulam cannot degrade RBM39, nor does it induce apoptosis in T-ALL tumor cells. These results suggest that the absence or low expression of DCAF15 in tumor cells is one of the mechanisms underlying the development of resistance to indisulam. Our research also revealed that in HUT78 and 6T-cem cells, the mRNA expression of DCAF15 is similar to that in other T-ALL cells, and RBM39 can be significantly degraded after treatment with indisulam. However, these two cell lines are resistant to indisulam, suggesting that the survival of some tumor cells does not depend on RBM39. Moreover, DCAF15 cannot fully predict the efficacy of the antitumor action of indisulam. Future research needs to focus on the mechanisms of resistance to indisulam and on finding better molecular markers that can predict the antitumor effects of indisulam. A 2020 study on biomarkers in AML proposed a novel biomarker by quantitatively assessing splice alterations using PCR. The study used indisulam-dependent splicing as a pharmacodynamic marker for treatment efficacy, a hypothesis that was validated in primary patient cells. Consequently, our research suggests that THOC1 may be considered a candidate factor for dependency splicing in future therapeutic applications [18].

In summary, our research identified a new candidate inhibitor for targeted therapy in T-ALL and validated RBM39 as an important survival-dependent gene in T-ALL. Through cross-analysis of RNA-seq and quantitative proteomics data, we found that the drug induced widespread RNA alternative splicing and downregulated the expression of related proteins, among which THOC1 and several other genes may be key factors involved in the antitumor effects of indisulam.

## Conclusions

In conclusion, our findings indicate that indisulam represents a potent therapeutic agent for T-ALL that functions through the targeted depletion of RBM39 to trigger tumor cell apoptosis. RBM39 has been identified as a critical oncogenic factor in T-ALL pathogenesis. The mechanism of action of indisulam involves the degradation of RBM39, precipitating extensive mis-splicing events in pre-RNA that disrupt normal translation processes, ultimately leading to the absence of the pivotal downstream effector THOC1.

### Supplementary Information

Supplementary Material 1: Supplementary Figure 1. Drug sensitivity assay of AML cell lines, including Kasumi-1,MV 4-11,K562 and U937, after treatment with gradient concentration of indisulam for 48 h.

Supplementary Material 2: Supplementary Figure 2. a. Indisulam induced cell cycle arrest, significantly increasing the proportion of cells in the G2 phase while reducing the proportion of cells in the G1 phase. b. The apoptotic rate of J.gamma1 cells was quantified and subjected to a statistical analysis 24 h after the administration of indisulam.

Supplementary Material 3: Supplementary Figure 3. Hematoxylin and eosin staining assessment of bone marrow, liver, and spleen in the two groups.

Supplementary Material 4: Supplementary Figure 4. FlowJo analysis demonstrated an elevation in of the proportion of apoptotic J.gamma1 and Jurkat cells following RBM39 knockdown.

Supplementary Material 5: Supplementary Figure 5. Representative immunohistochemical staining images of mouse bone marrow. Immunohistochemistry was used to determine RBM39-positive regions and Ki67-positive regions.

Supplementary Material 6: Supplementary Figure 6. IGV displays widespread mis-splicing events after treatment with indisulam, with EZH2, TYMS, THOC1, THOC5, and CDC25C serving as examples.

Supplementary Material 7: Supplementary Figure 7. a. The knockdown of THOC1 affects cell colony formation and proliferation. b. Flow cytometry analysis indicated that the depletion of THOC1 led to a notable increase in cellular apoptosis.

Supplementary Material 8: Supplementary Figure 8. Flow cytometric analysis was performed to assess apoptosis in DCAF15^KO^ and DCAF15^WT^ cells after 48 h of treatment with DMSO or different concentrations of indisulam using Annexin V and PI staining. The percentage of apoptotic cells was statistically evaluated.

Supplementary Material 9: Supplementary Figure 9. a. After treatment with indisulam for 24 and 48 h, Western blot assays were conducted to assess alterations in RBM39 and THOC1 protein expression within the 6T-CEM and HUT78 cell lines. b. PCR confirmation of DCAF15 mRNA expression levels in six T-ALL cell lines.

Supplementary Material 10: Supplementary Table 1. Primer sequence information, plasmid sequence information, antibody information.

Supplementary Material 11: Supplementary Table 2. The area under the curve (AUC) reflecting the sensitivity of the drug indisulam across different tissue tumors within the CTD2 network.

Supplementary Material 12: Supplementary Table 3. The relative expression levels of RBM39 in T-ALL data from GSE110637 and normal blood data from the GTEx.

Supplementary Material 13: Supplementary Table 4. Exon events occurring in RNA following indisulam treatment.

Supplementary Material 14: Supplementary Table 5. Intron events occurring in RNA following indisulam treatment.

Supplementary Material 15: Supplementary Table 6. Mutually exclusive exons events occurring in RNA following indisulam treatment.

Supplementary Material 16: Supplementary Table 7. Alternative 5’ splice site events occurring in RNA following indisulam treatment.

Supplementary Material 17: Supplementary Table 8. Alternative 3’ splice site events occurring in RNA following indisulam treatment.

Supplementary Material 18: Supplementary Table 9. Genes with RNA mis-splicing and differential protein quantification overlap.

Supplementary Material 19: Supplementary Table 10. Genes exhibiting concurrence between mis-splicing events and downregulated proteins identified via LC/MS and the enriched KEGG pathways.

Supplementary Material 20: Supplementary Table 11. All proteins exhibiting differential expression as identified by LC/MS analysis.

Supplementary Material 21: Supplementary Table 12. CRISPR screening data indicated the dependence of Jurkat cell survival on THOC1.

Supplementary Material 22.

Supplementary Material 23.

## Data Availability

The RNA-seq data used and/or analyzed during the current study are available from the corresponding authors on reasonable request (GSE263309).

## Abbreviations

T-ALL: T-cell acute lymphoblastic leukemia
CNS: Central nervous system
RBM39: RNA binding motif protein 39
shRNA: Short hairpin RNAs
ALL: Acute lymphoblastic leukemia
IMiDs: Immunomodulatory drugs
HCC1: Hepato-cellular carcinoma 1
CAPER or CAPERα: Coactivator of activating protein-1 [AP-1] and oestrogen receptors [ERs]
RBP: RNA binding protein
AML: Acute myeloid leukemia
RPMI: Roswell Park Memorial Institute
DMEM: Dulbecco’s modified Eagle’s
IC50: The half-maximum inhibitory concentration
CCK8: Cell Counting Kit 8
RIPA buffer: Radio Immunoprecipitation Assay Lysis buffer
PVDF: Polyvinylidene fluoride
TBST: Tris buffered saline with Tween-20
PEI: Polyethylenimine
NSG: NOD scid gamma
qRT-PCR: Quantitative real-time PCR
PCR: Polymerase chain reaction
sgRNAs: Small guide oligos
RNA-seq: RNA sequencing
HL: Hematopoietic and lymphoid
AUC: Area-under-the-curve
sh-RBM39: ShRNA targeting RBM39
sh-THOC1: ShRNA targeting THOC1
LC–MS/MS: Liquid chromatography tandem mass spectrometry
