# Supplementary figures and images for: Targeting RBM39 through indisulam induced mis-splicing of mRNA to exert anti-cancer effects in T-cell acute lymphoblastic leukemia
Source: J Exp Clin Cancer Res. 2024 Jul 24;43:205. doi: 10.1186/s13046-024-03130-8 (PMC11267830; doi:10.1186/s13046-024-03130-8)

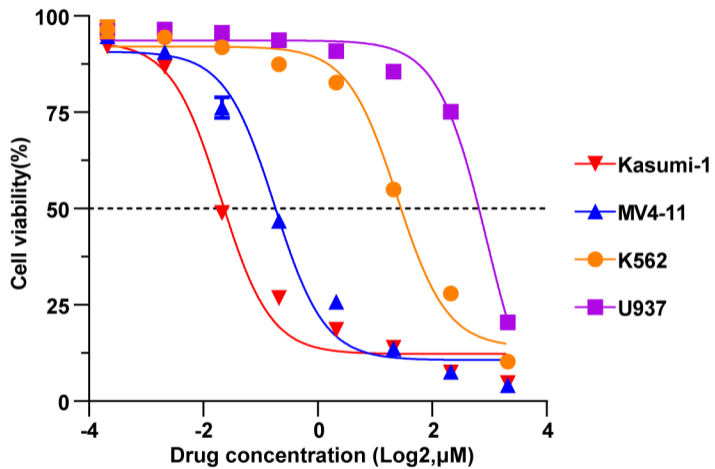

| Cell Line | IC50(μM) | 95%CI               |
|-----------|----------|---------------------|
| Kasumi-1  | 0.02005  | 0.001387 to 0.02941 |
| MV-4-11   | 0.1760   | 0.1164 to 0.2651    |
| K-562     | 24.35    | 17.67 to 33.68      |
| U-937     | 921.1    | 635.1 to 1488       |

Supplement: Supplementary file 1 — Supplementary Material 1: Supplementary Figure 1. Drug sensitivity assay of AML cell lines, including Kasumi-1,MV 4-11,K562 and U937, after treatment with gradient concentration of indisulam for 48 h. [file 13046_2024_3130_MOESM1_ESM.pdf]

**a**

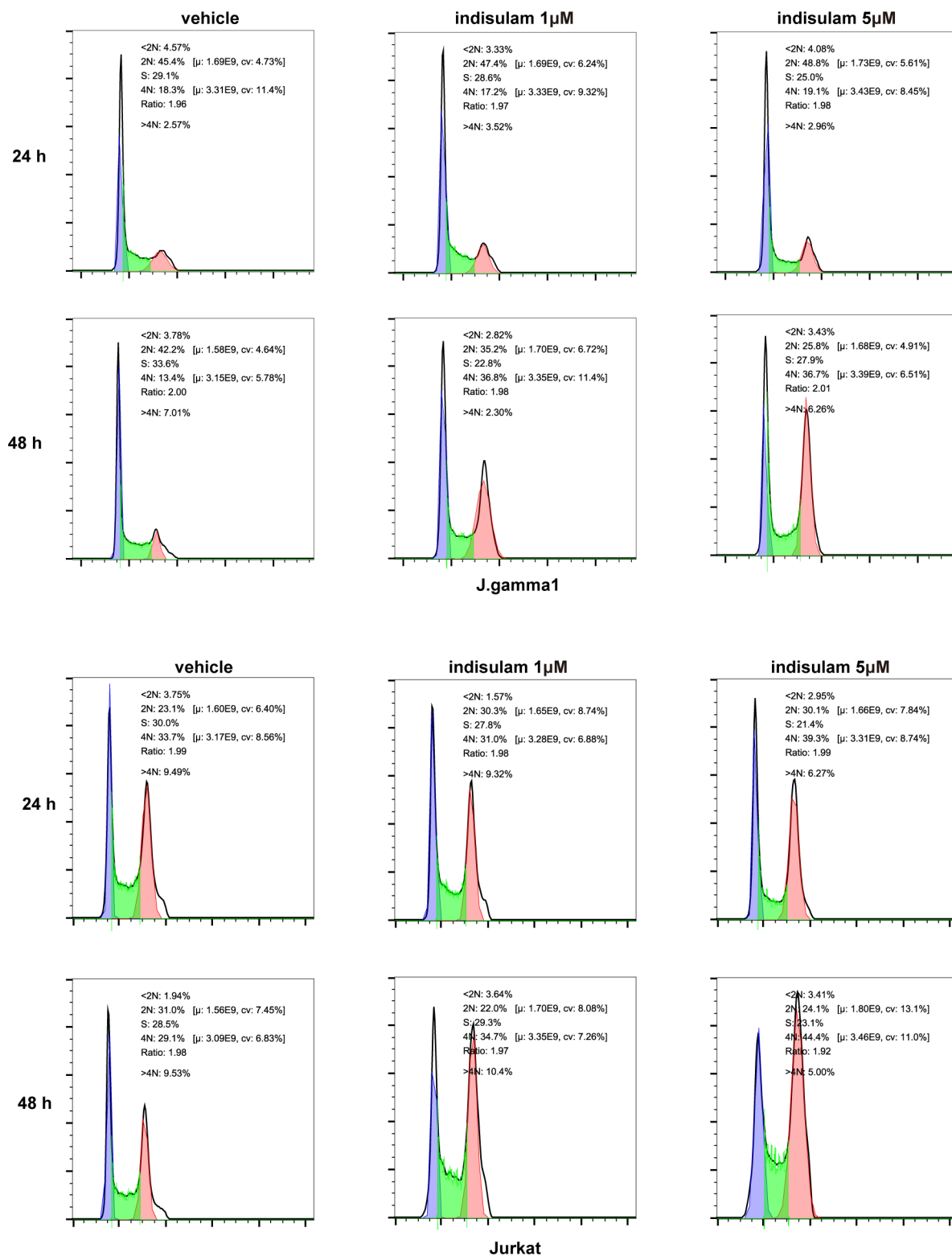

**b**

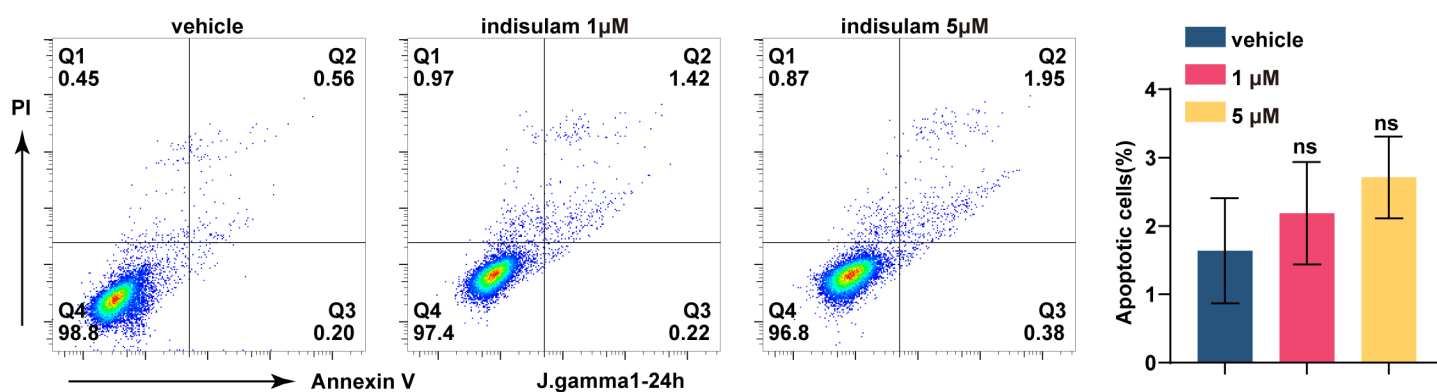

Supplement: Supplementary file 2 — Supplementary Material 2: Supplementary Figure 2. a. Indisulam induced cell cycle arrest, significantly increasing the proportion of cells in the G2 phase while reducing the proportion of cells in the G1 phase. b. The apoptotic rate of J.gamma1 cells was quantified and subjected to a statistical analysis 24 h after the administration of indisulam. [file 13046_2024_3130_MOESM2_ESM.pdf]

### HE-Bone Marrow

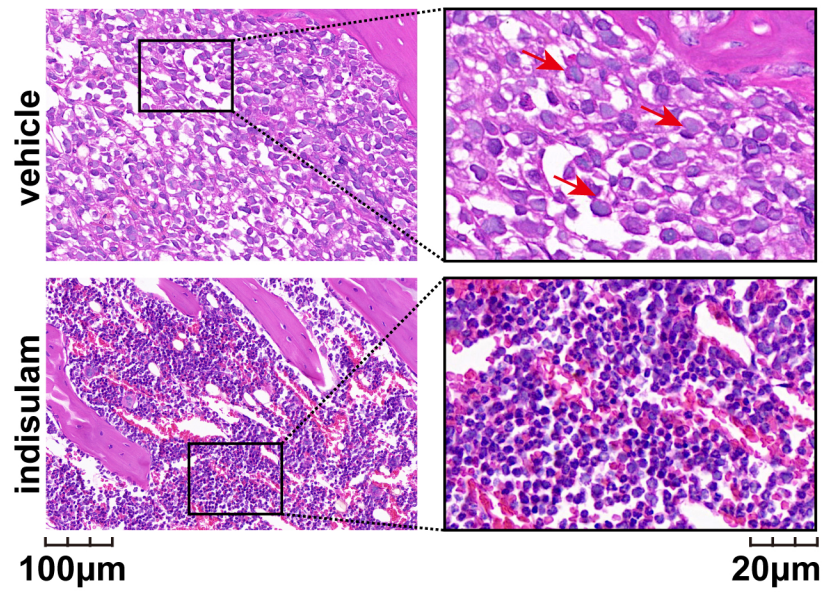

### HE-Liver

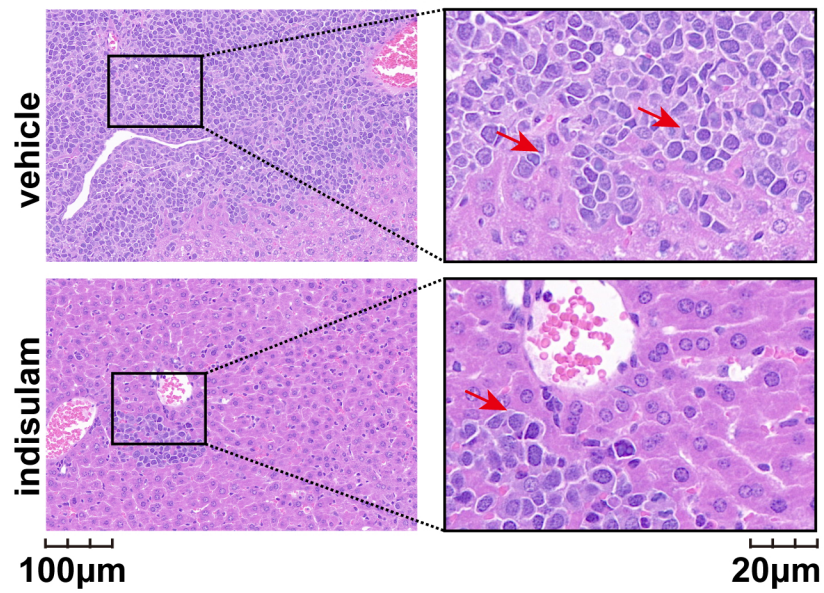

### HE-Spleen

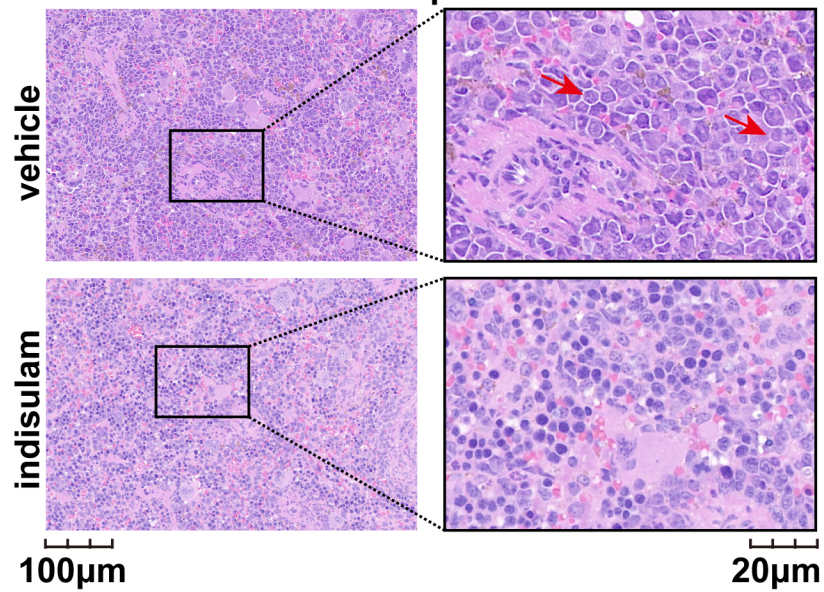

Supplement: Supplementary file 3 — Supplementary Material 3: Supplementary Figure 3. Hematoxylin and eosin staining assessment of bone marrow, liver, and spleen in the two groups. [file 13046_2024_3130_MOESM3_ESM.pdf]

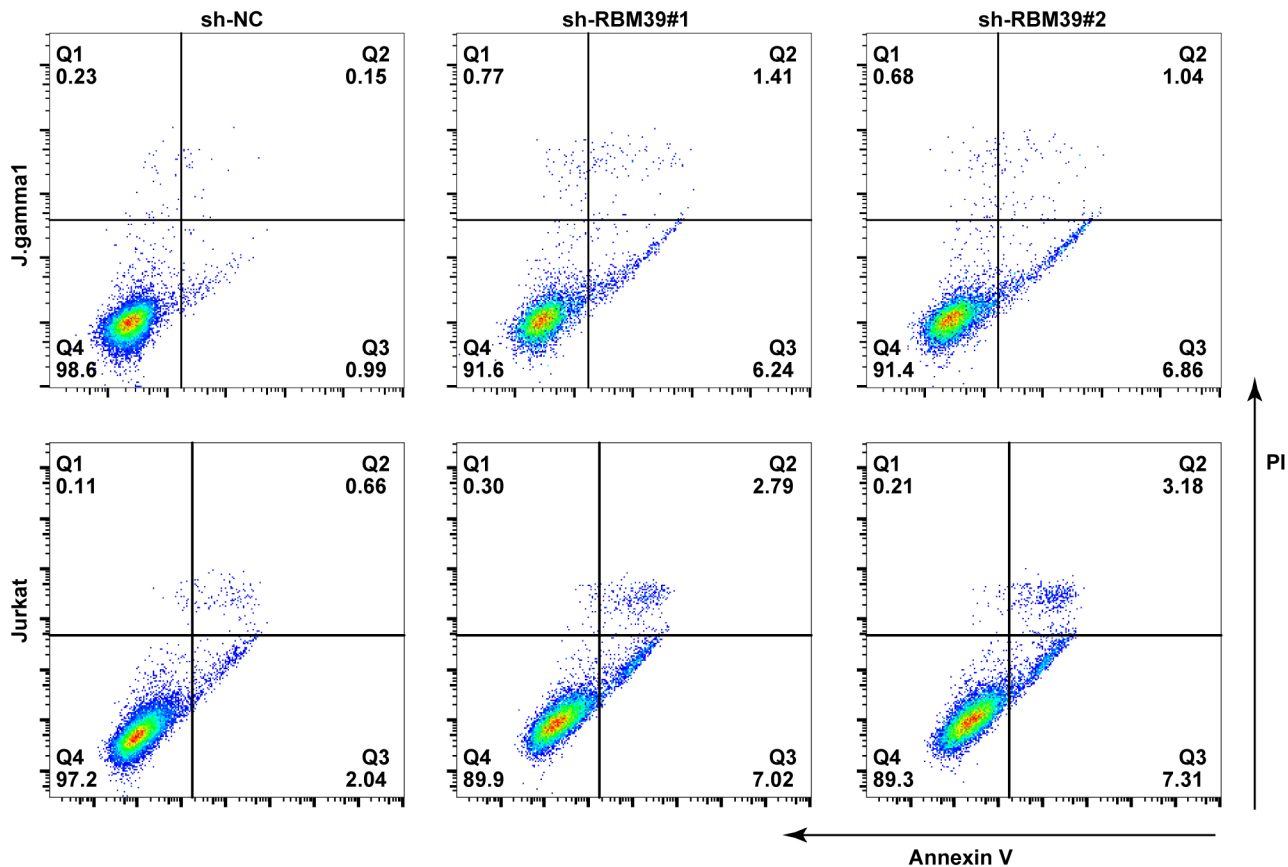

Supplement: Supplementary file 4 — Supplementary Material 4: Supplementary Figure 4. FlowJo analysis demonstrated an elevation in of the proportion of apoptotic J.gamma1 and Jurkat cells following RBM39 knockdown. [file 13046_2024_3130_MOESM4_ESM.pdf]

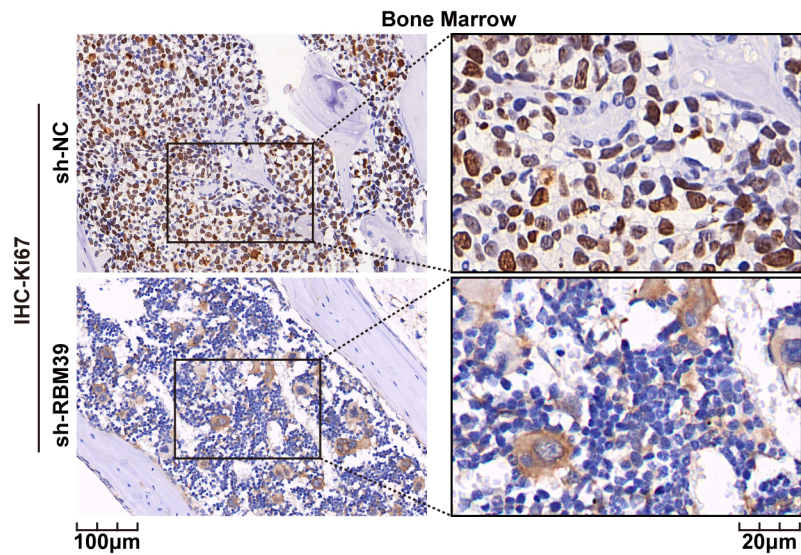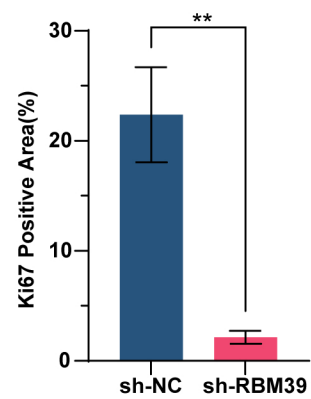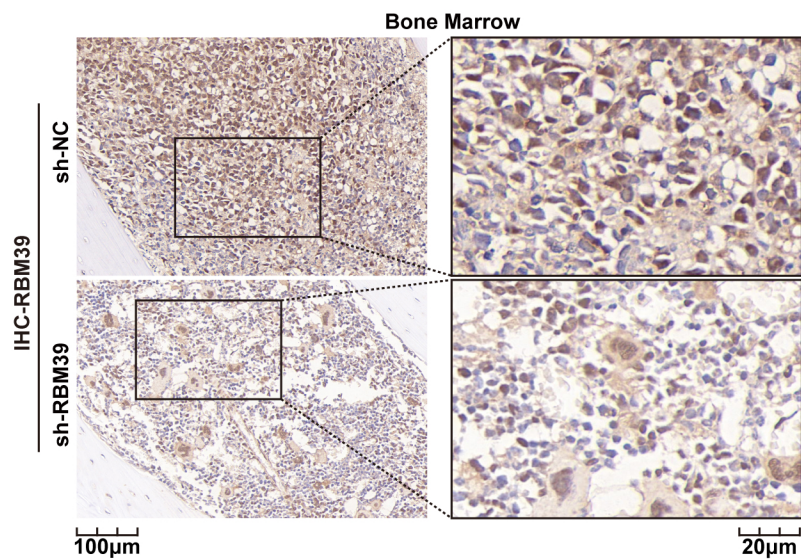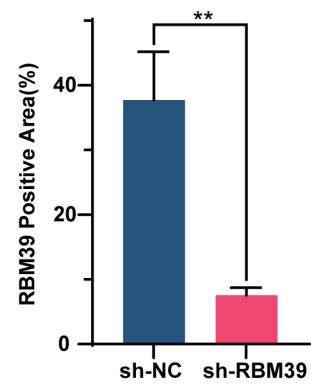

Supplement: Supplementary file 5 — Supplementary Material 5: Supplementary Figure 5. Representative immunohistochemical staining images of mouse bone marrow. Immunohistochemistry was used to determine RBM39-positive regions and Ki67-positive regions. [file 13046_2024_3130_MOESM5_ESM.pdf]

EZH2

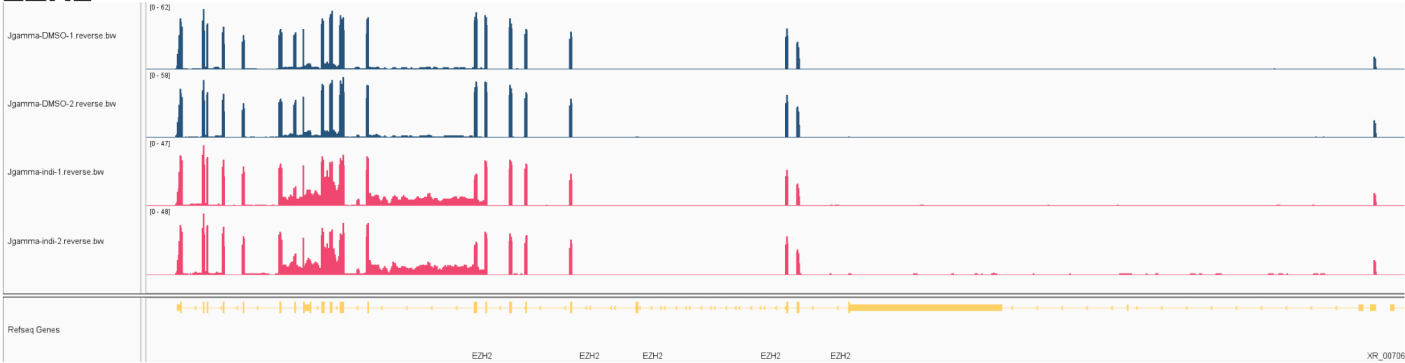

THOC1

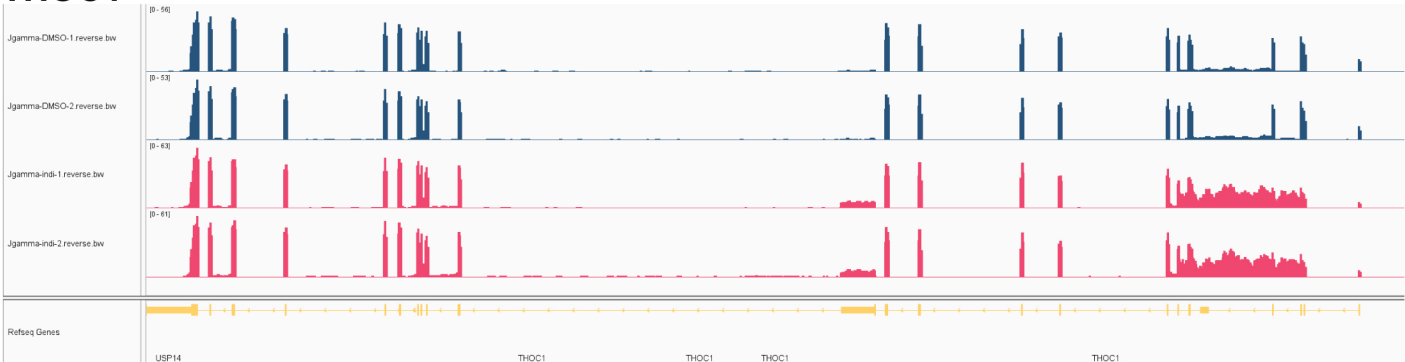

TYMS

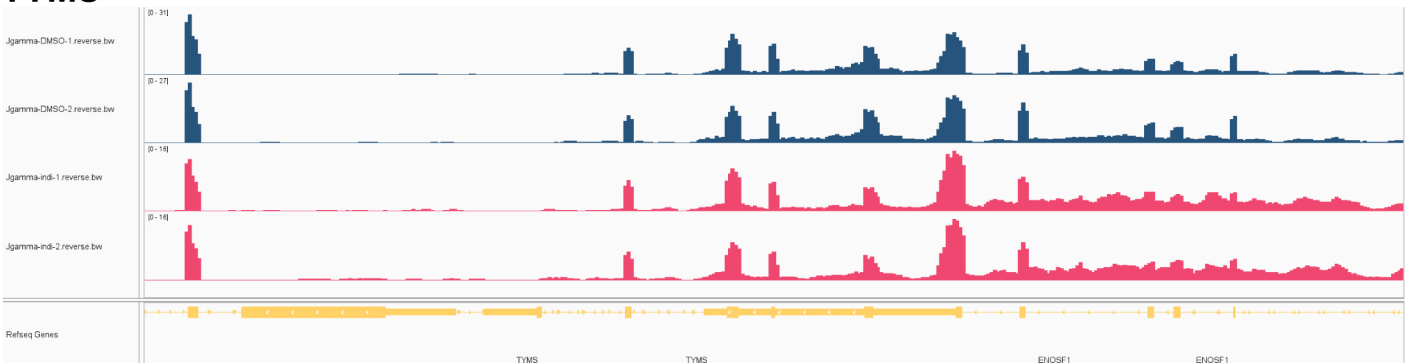

CDC25C

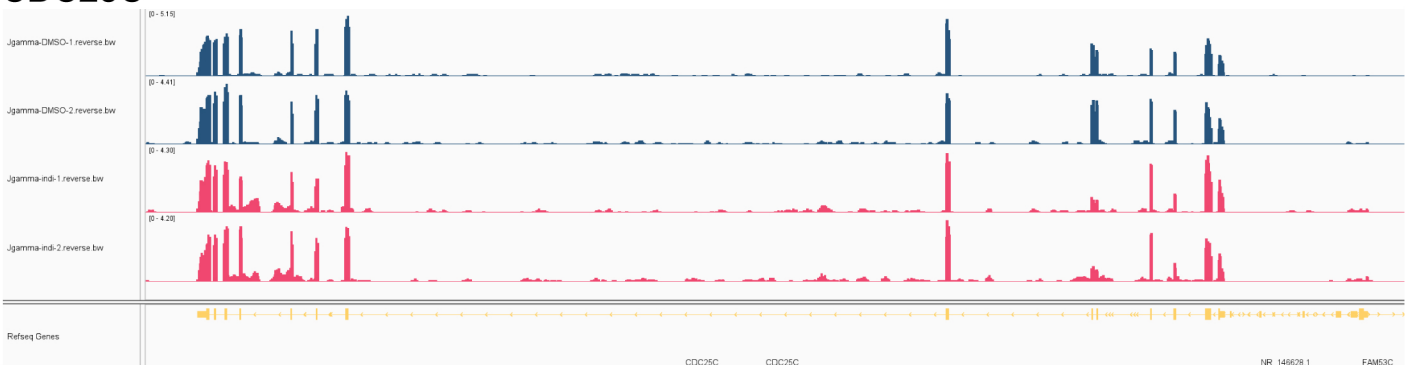

THOC5

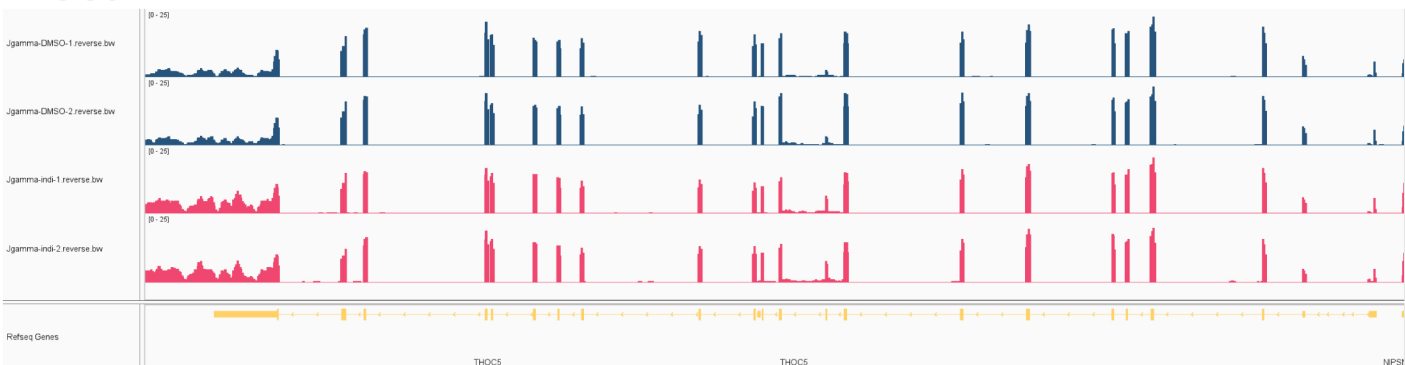

Supplement: Supplementary file 6 — Supplementary Material 6: Supplementary Figure 6. IGV displays widespread mis-splicing events after treatment with indisulam, with EZH2, TYMS, THOC1, THOC5, and CDC25C serving as examples. [file 13046_2024_3130_MOESM6_ESM.pdf]

**a**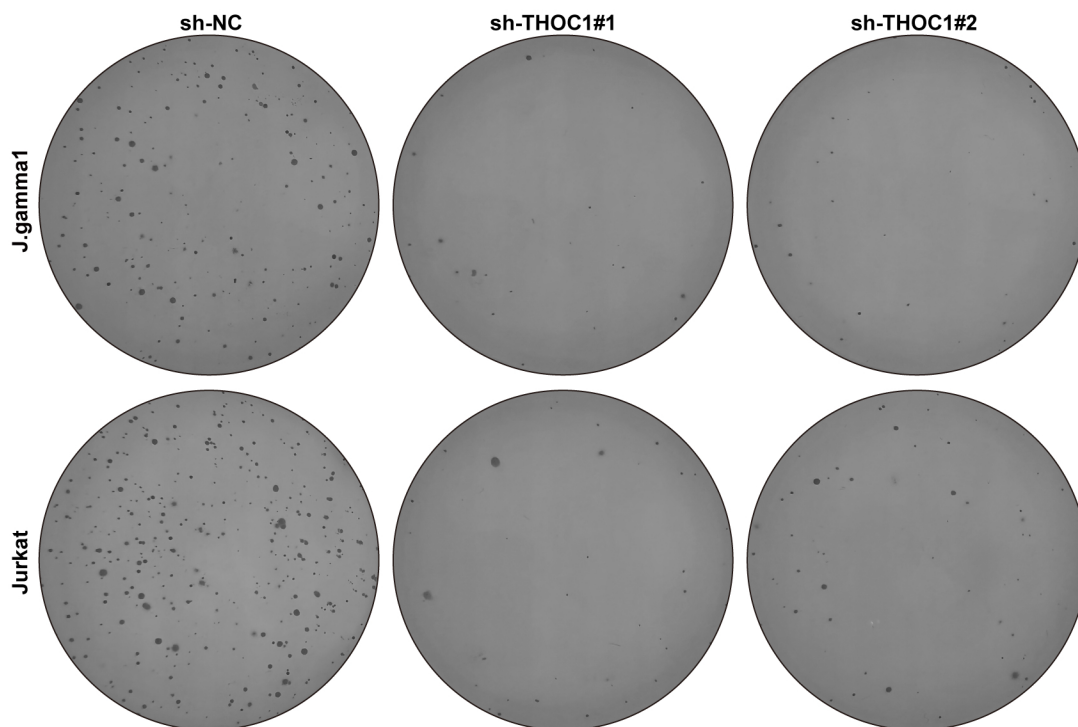**b**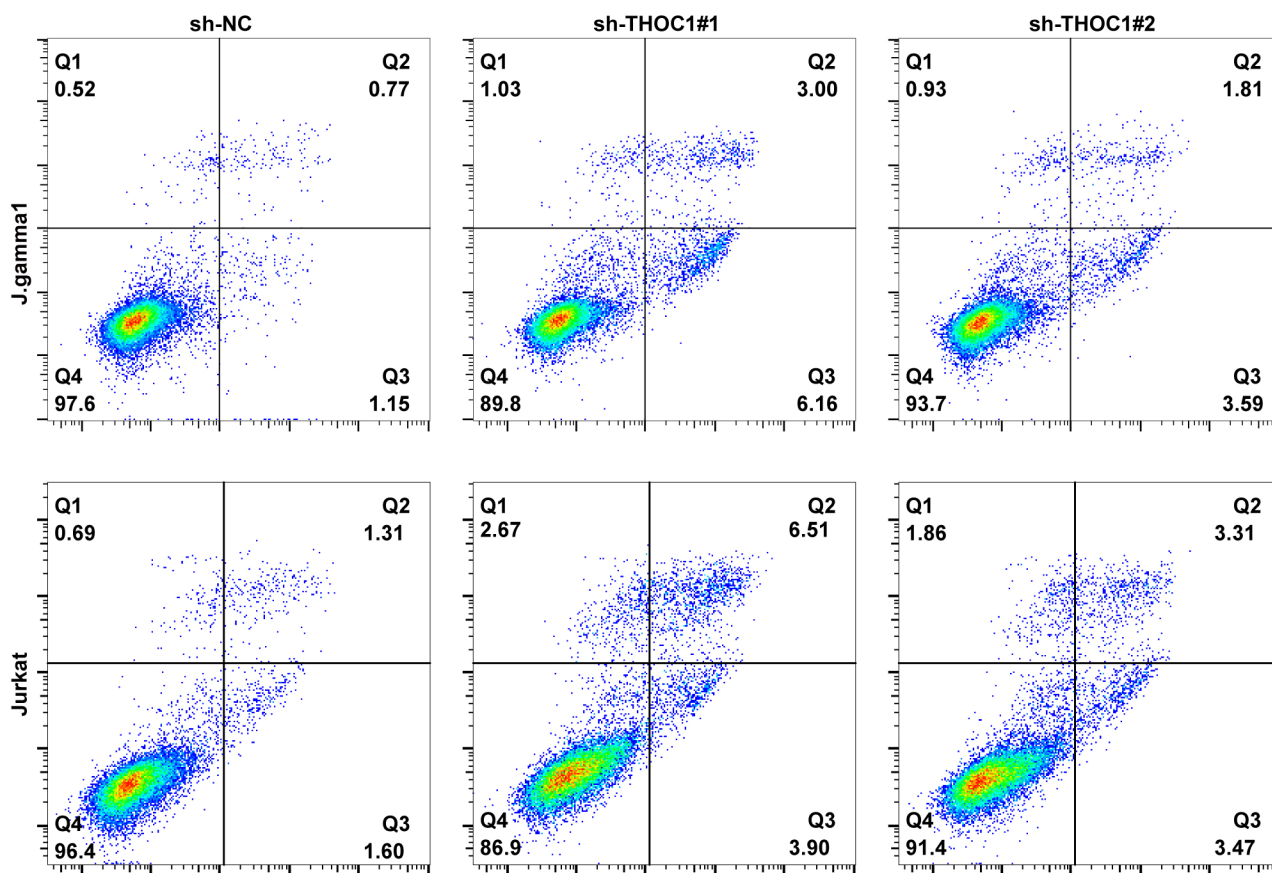

Supplement: Supplementary file 7 — Supplementary Material 7: Supplementary Figure 7. a. The knockdown of THOC1 affects cell colony formation and proliferation. b. Flow cytometry analysis indicated that the depletion of THOC1 led to a notable increase in cellular apoptosis. [file 13046_2024_3130_MOESM7_ESM.pdf]

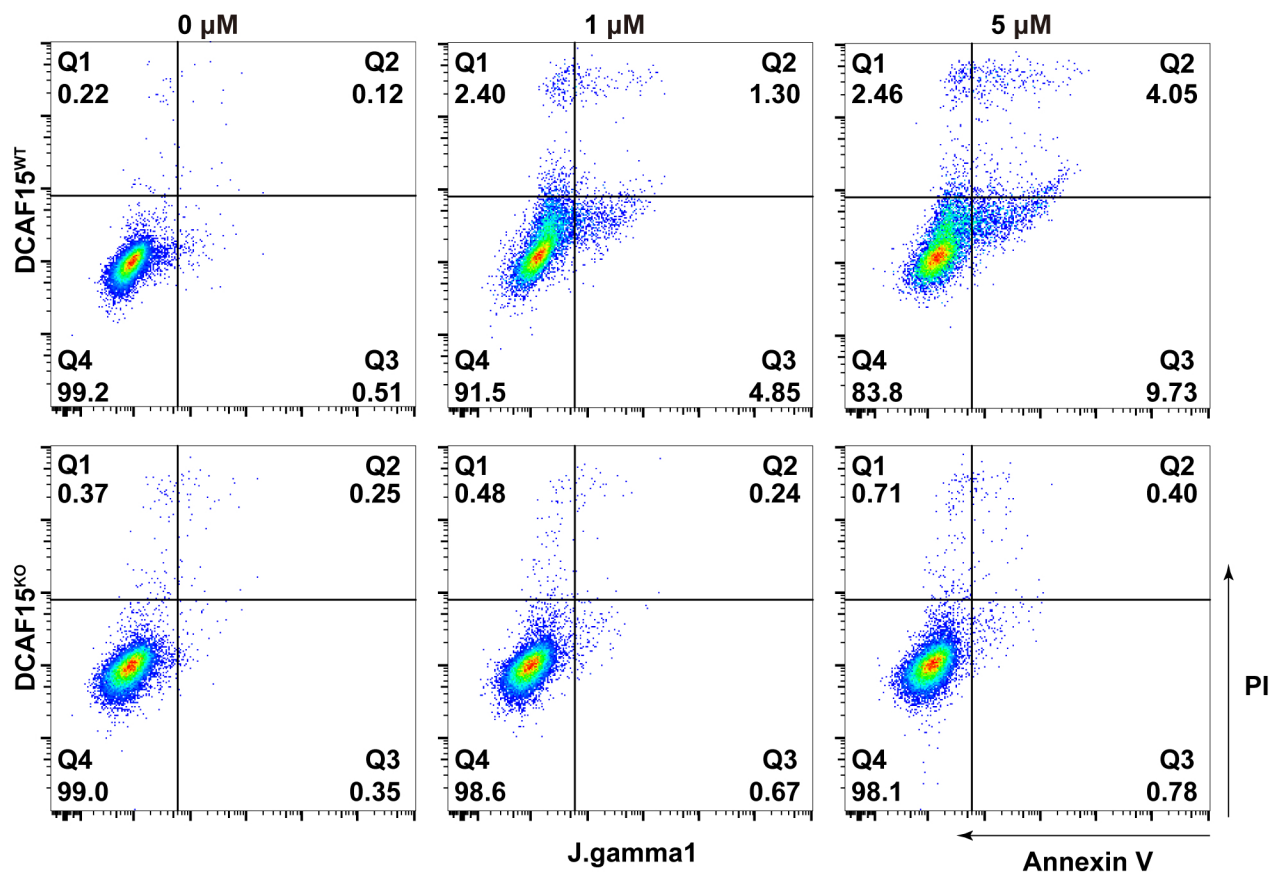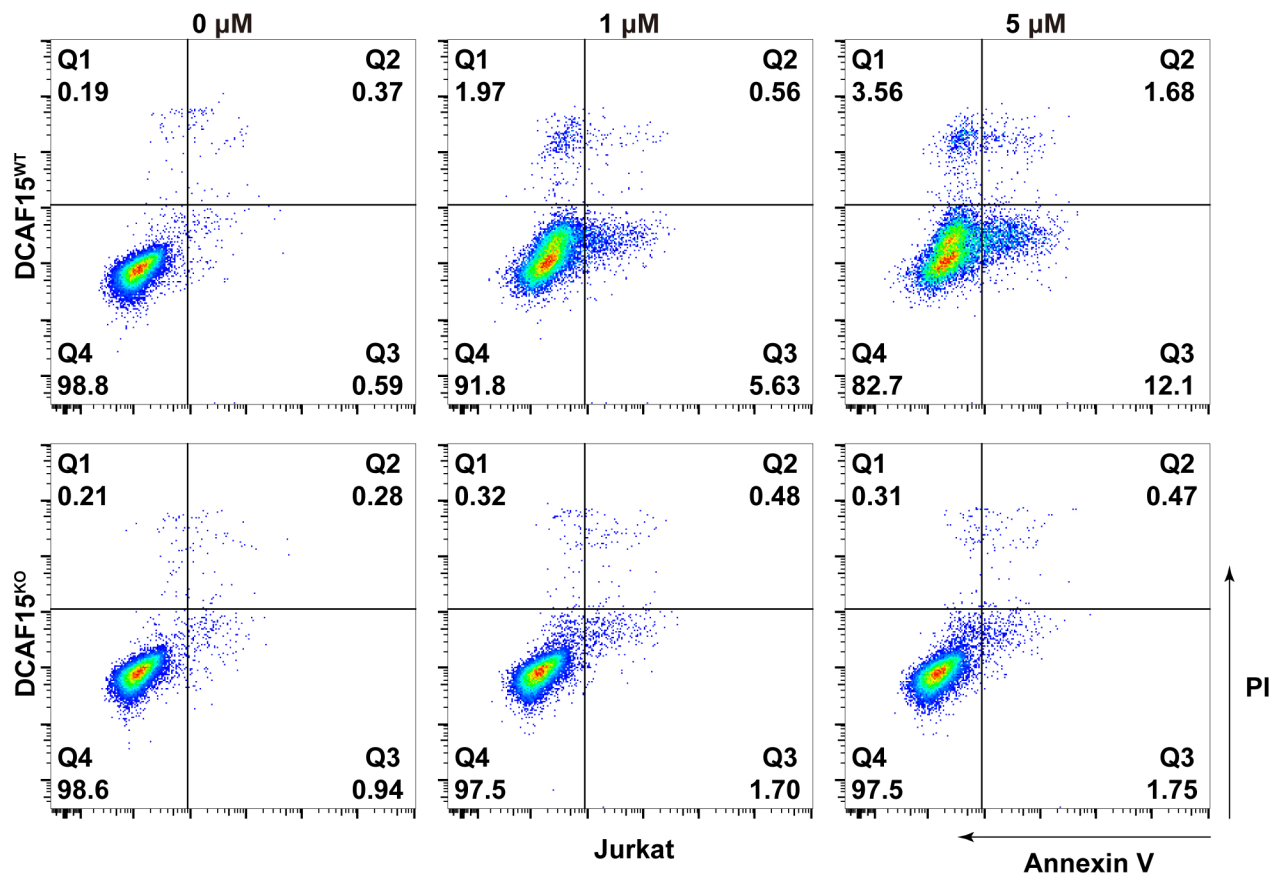

Supplement: Supplementary file 8 — Supplementary Material 8: Supplementary Figure 8. Flow cytometric analysis was performed to assess apoptosis in DCAF15KO and DCAF15WT cells after 48 h of treatment with DMSO or different concentrations of indisulam using Annexin V and PI staining. The percentage of apoptotic cells was statistically evaluated. [file 13046_2024_3130_MOESM8_ESM.pdf]

**a**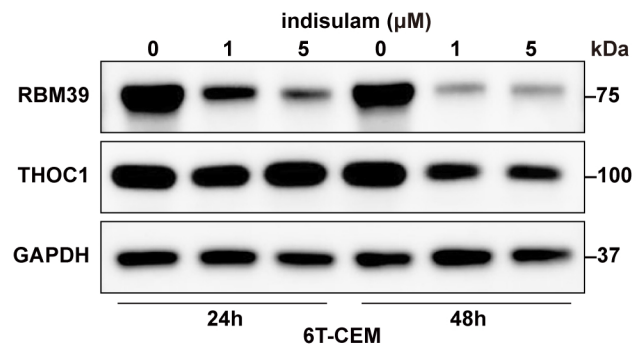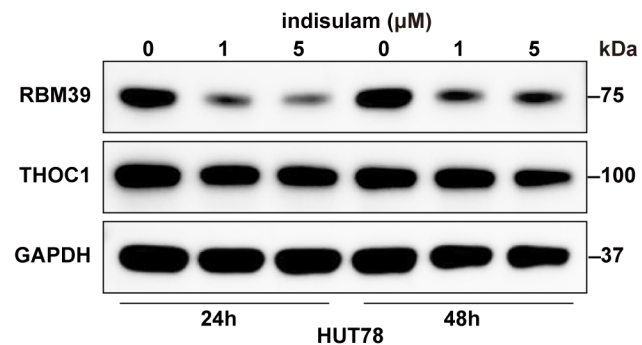**b**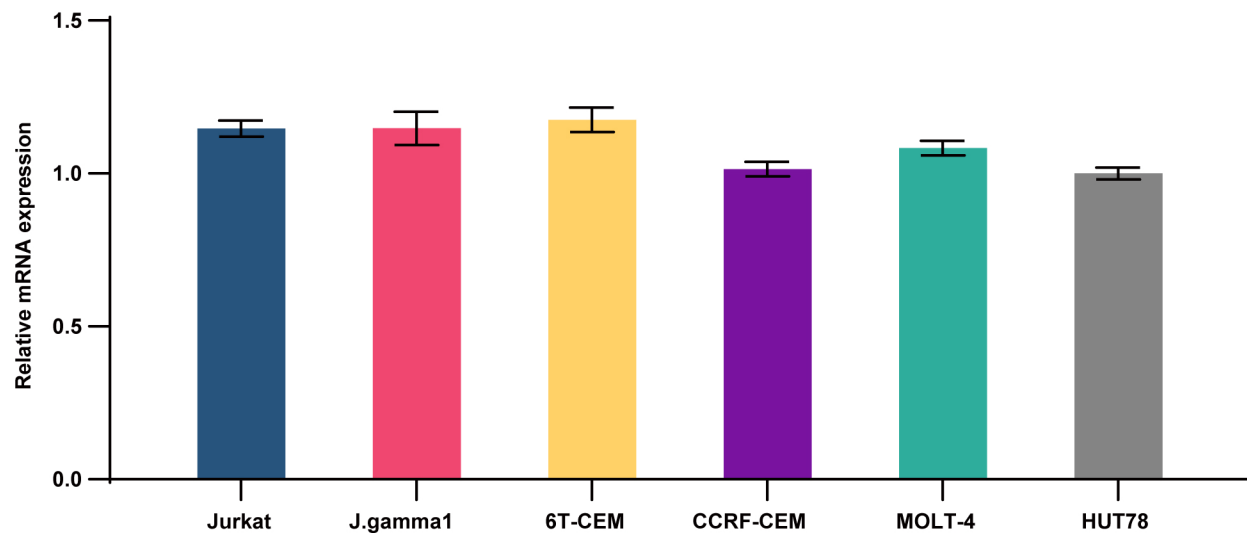

Supplement: Supplementary file 9 — Supplementary Material 9: Supplementary Figure 9. a. After treatment with indisulam for 24 and 48 h, Western blot assays were conducted to assess alterations in RBM39 and THOC1 protein expression within the 6T-CEM and HUT78 cell lines. b. PCR confirmation of DCAF15 mRNA expression levels in six T-ALL cell lines. [file 13046_2024_3130_MOESM9_ESM.pdf]
