# Supplementary material for: Targeting RBM39 through indisulam induced mis-splicing of mRNA to exert anti-cancer effects in T-cell acute lymphoblastic leukemia
Source: J Exp Clin Cancer Res. 2024 Jul 24;43:205. doi: 10.1186/s13046-024-03130-8 (PMC11267830; doi:10.1186/s13046-024-03130-8)

Figure 2e

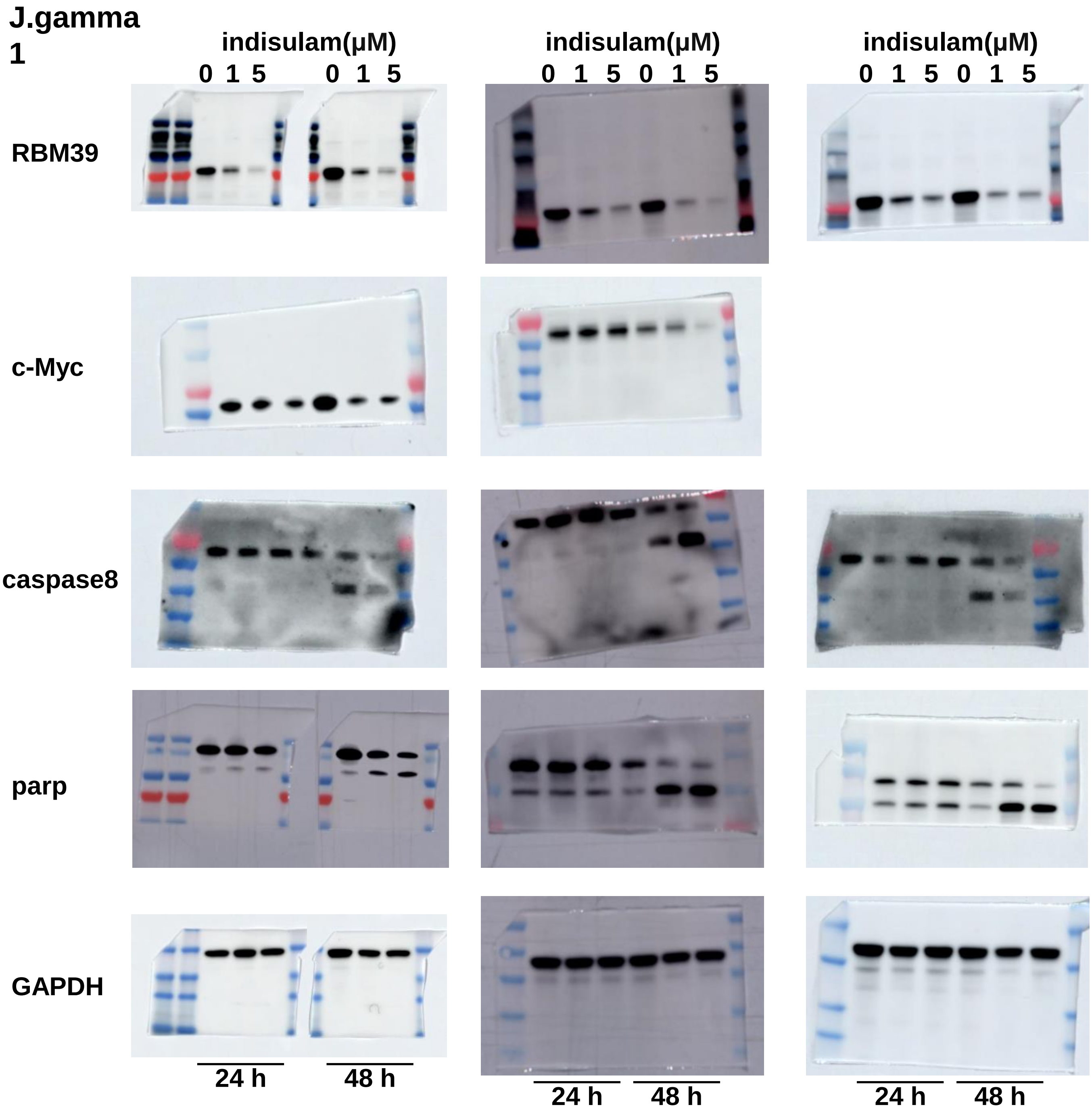
J.gamma 1
indisulam(μM)
0 1 5 0 1 5
indisulam(μM)
0 1 5 0 1 5
indisulam(μM)
0 1 5 0 1 5
RBM39
c-Myc
caspase8
parp
GAPDH
24 h
48 h
24 h
48 h
24 h
48 h

Figure 2e

Jurkat

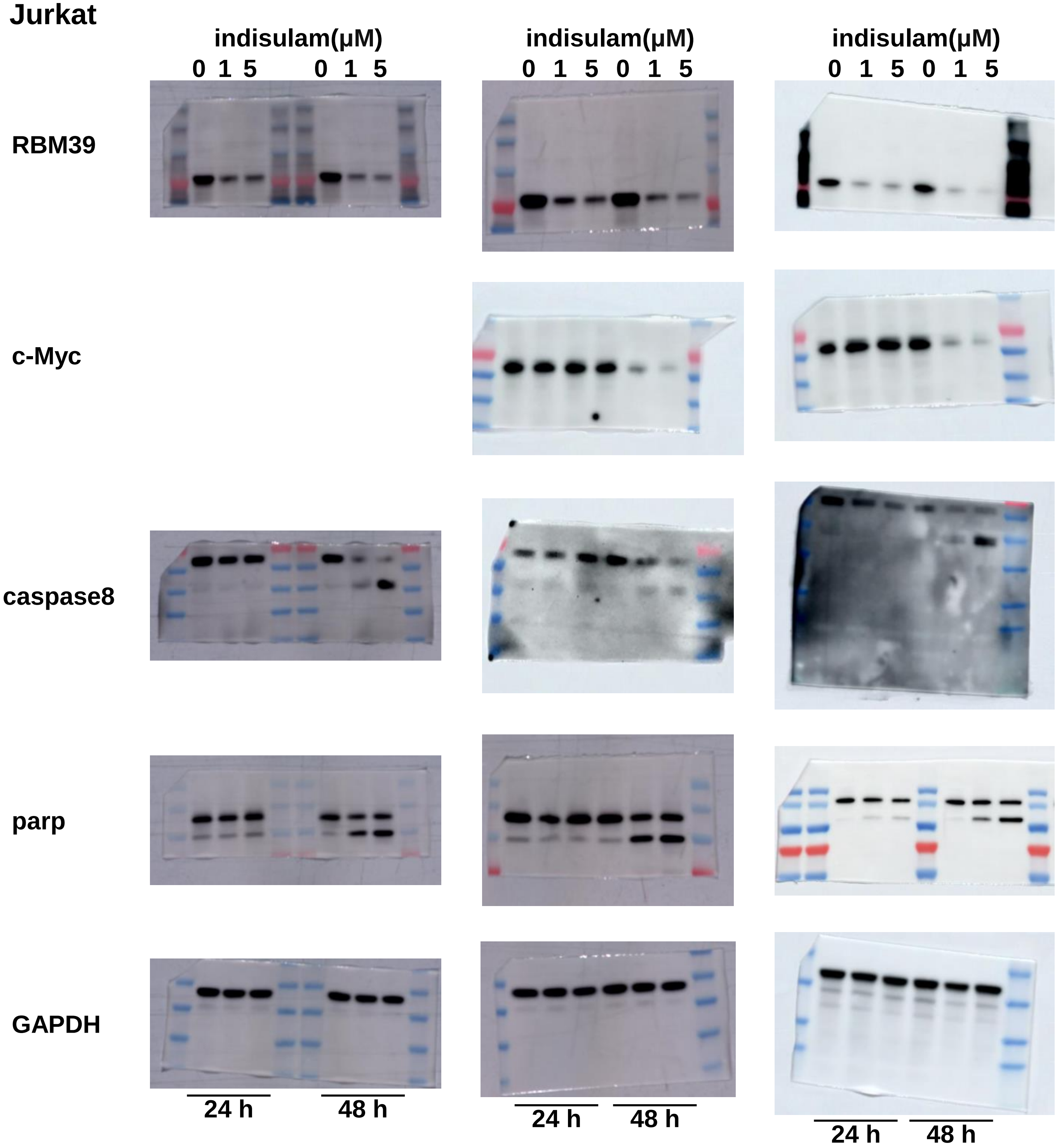

Figure 4b

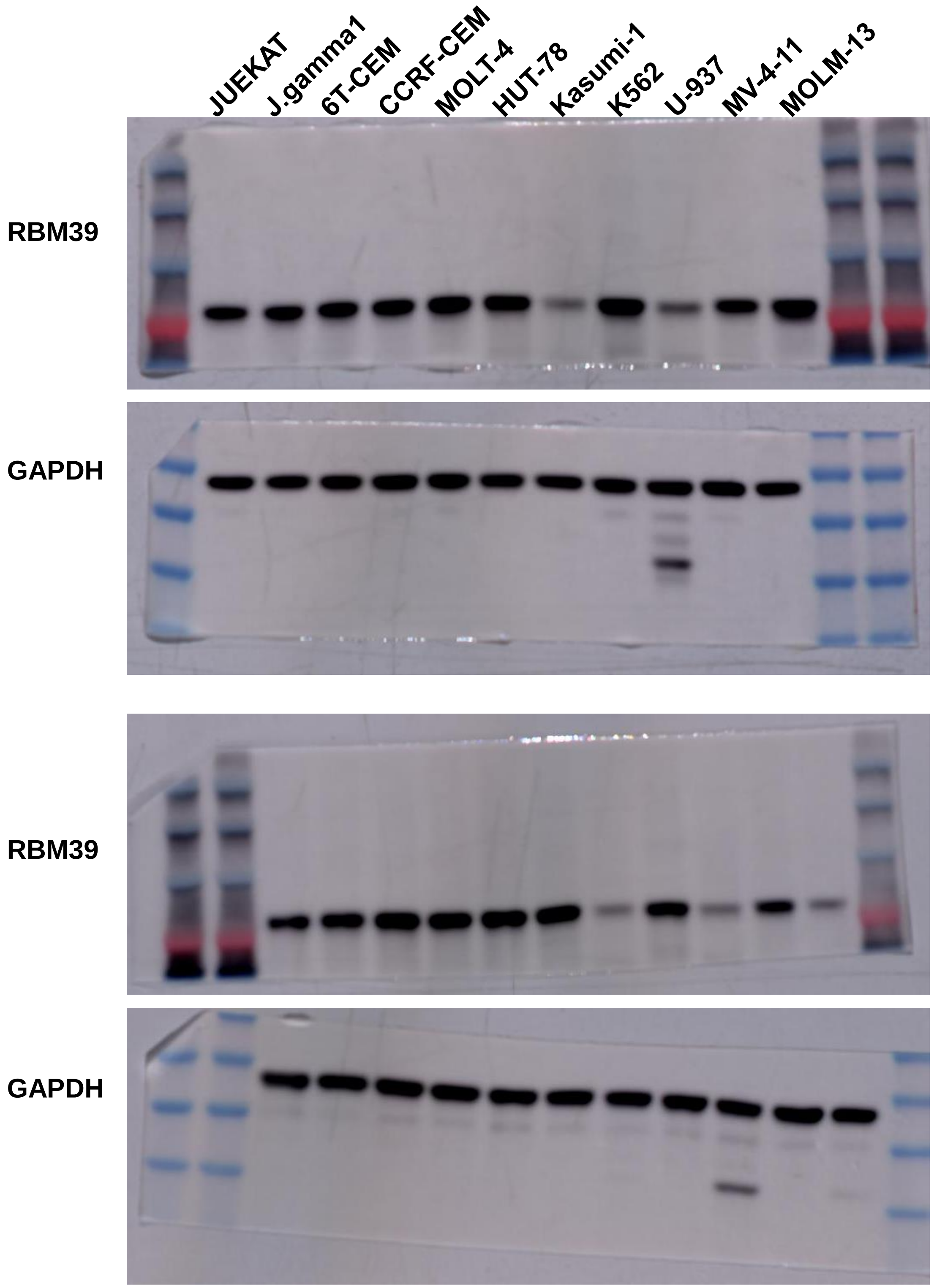
JUEKAT
J.gamma1
6T-CEM
CCRF-CEM
MOLT-4
HUT-78
Kasumi-1
K562
U-937
MV-4-11
MOLM-13
RBM39
GAPDH
RBM39
GAPDH

Figure 4c

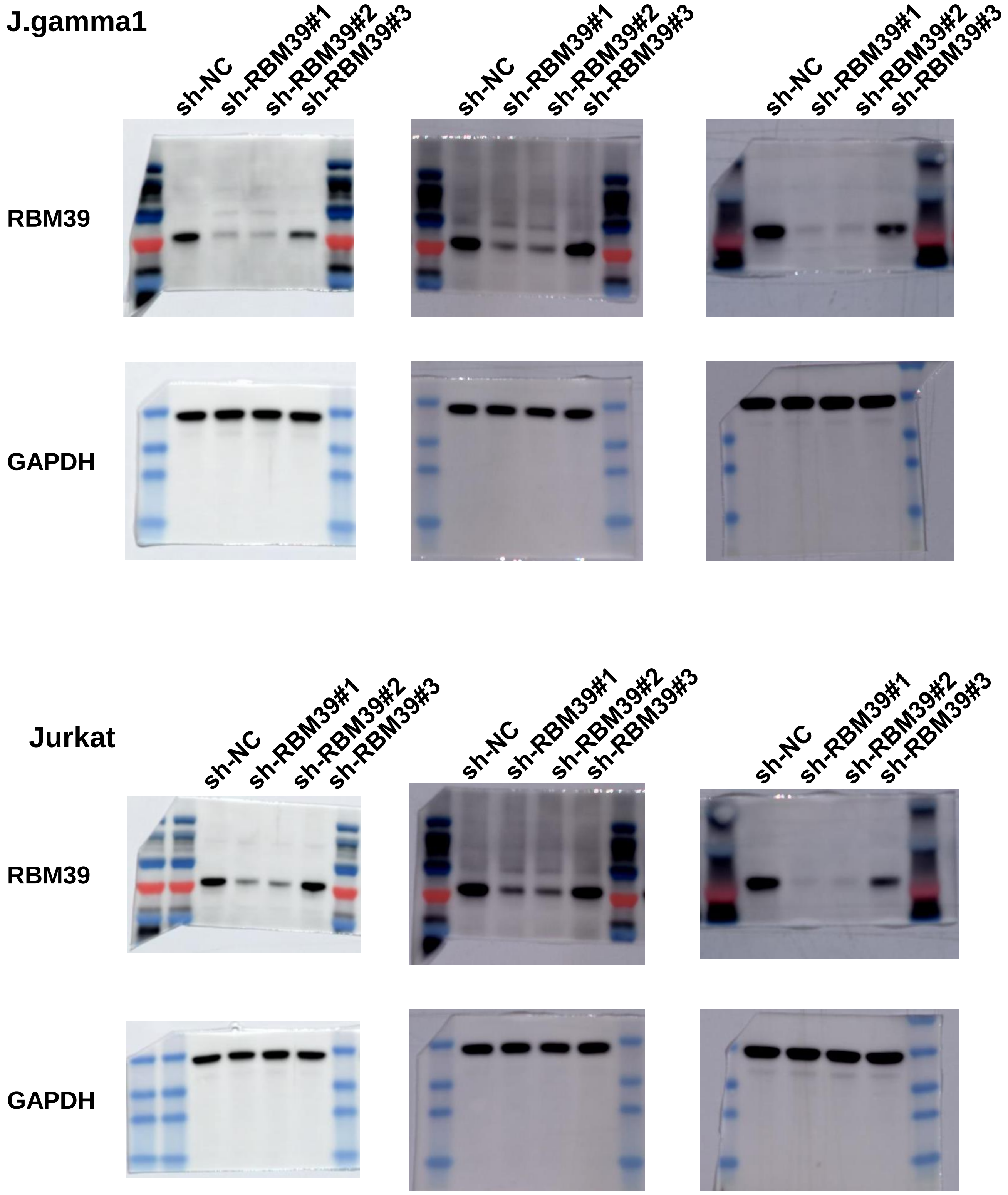
J.gamma1
sh-NC
sh-RBM39#1
sh-RBM39#2
sh-RBM39#3
RBM39
GAPDH
Jurkat
sh-NC
sh-RBM39#1
sh-RBM39#2
sh-RBM39#3
RBM39
GAPDH

Figure 4h

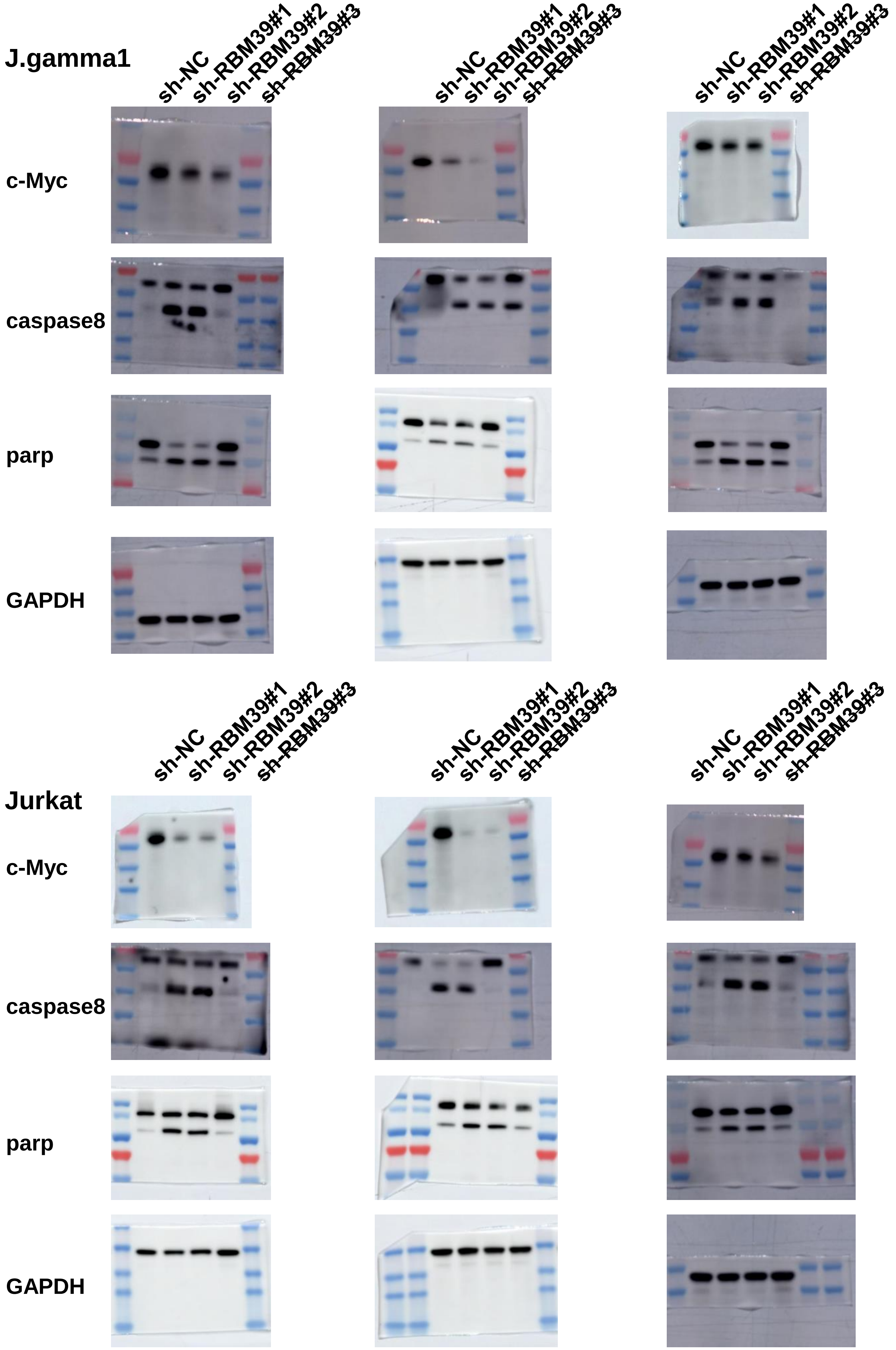

J.gamma1
sh-NC
sh-RBM39#1
sh-RBM39#2
sh-RBM39#3
c-Myc
caspase8
parp
GAPDH
Jurkat

Figure 6i

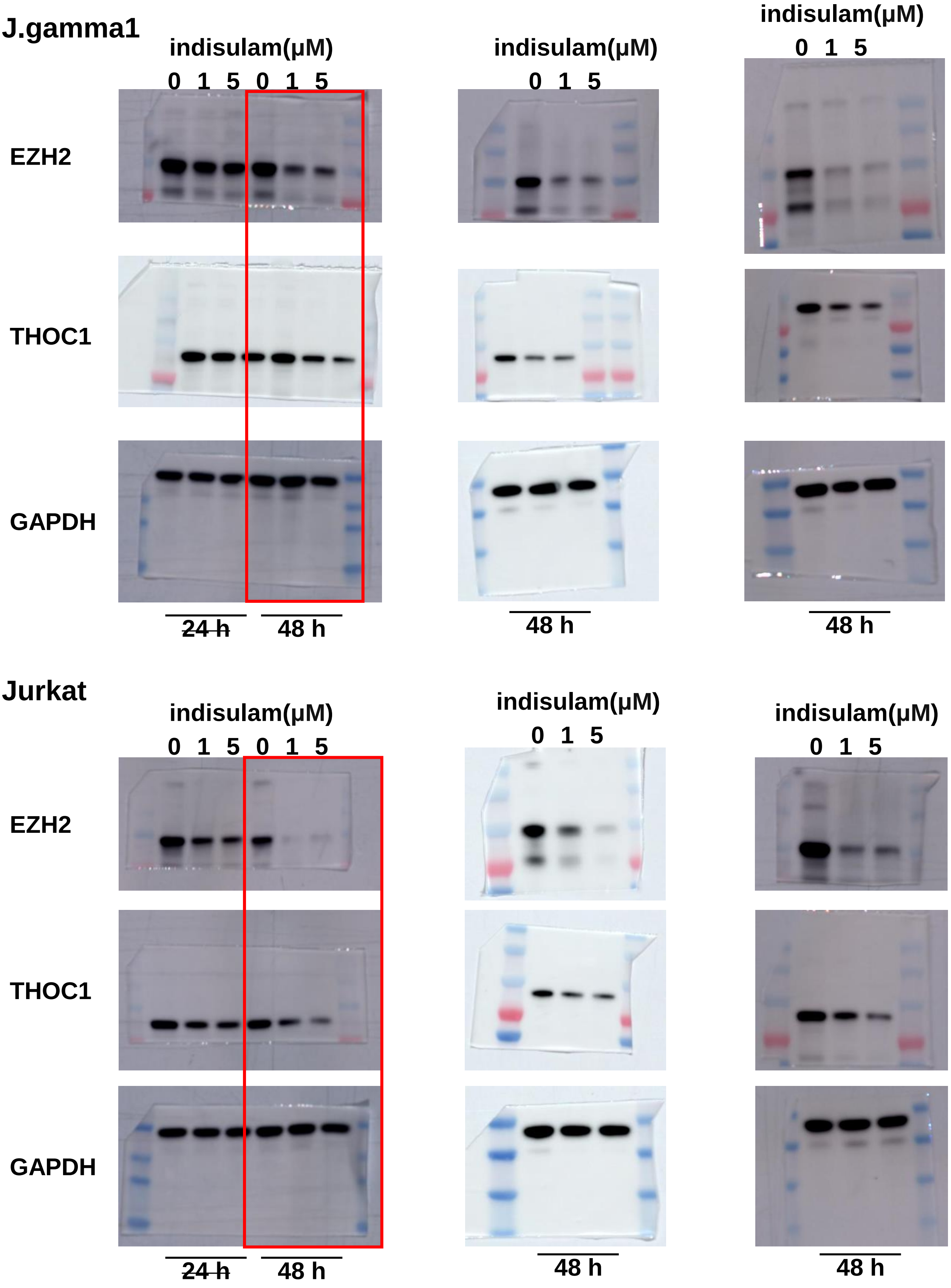
J.gamma1
indisulam(μM)
0 1 5 0 1 5
EZH2
THOC1
GAPDH
24 h
48 h
indisulam(μM)
0 1 5
48 h
indisulam(μM)
0 1 5
48 h
Jurkat
indisulam(μM)
0 1 5 0 1 5
EZH2
THOC1
GAPDH
24 h
48 h
indisulam(μM)
0 1 5
48 h
indisulam(μM)
0 1 5
48 h

Figure 7b

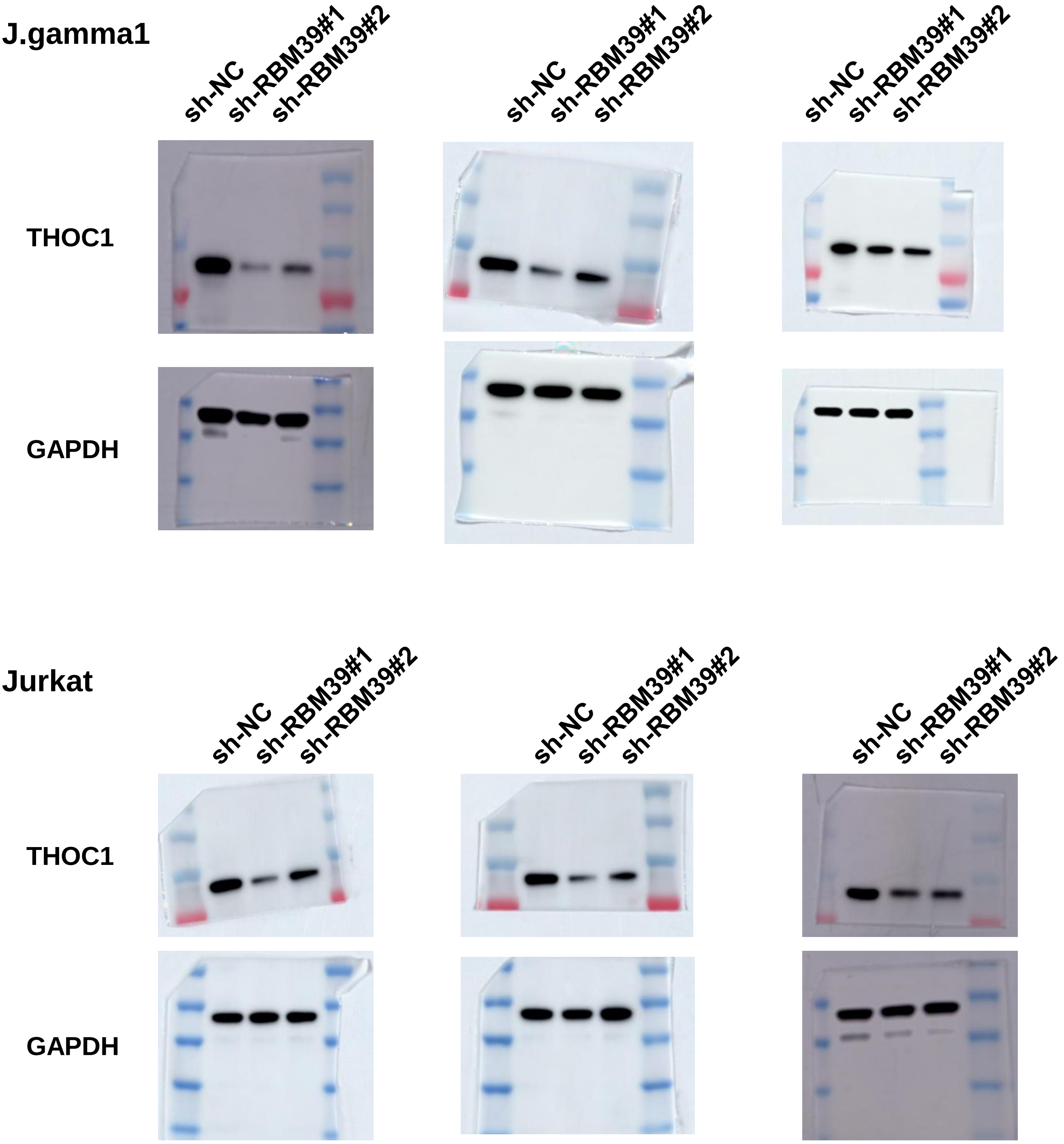

The sample here is the same as before. Since the positions of RBM39 and THOC1 proteins are close, and the interference efficiency of RBM39 has been verified in the experiment shown in Figure 4c, it will not be validated again.

Figure 7e

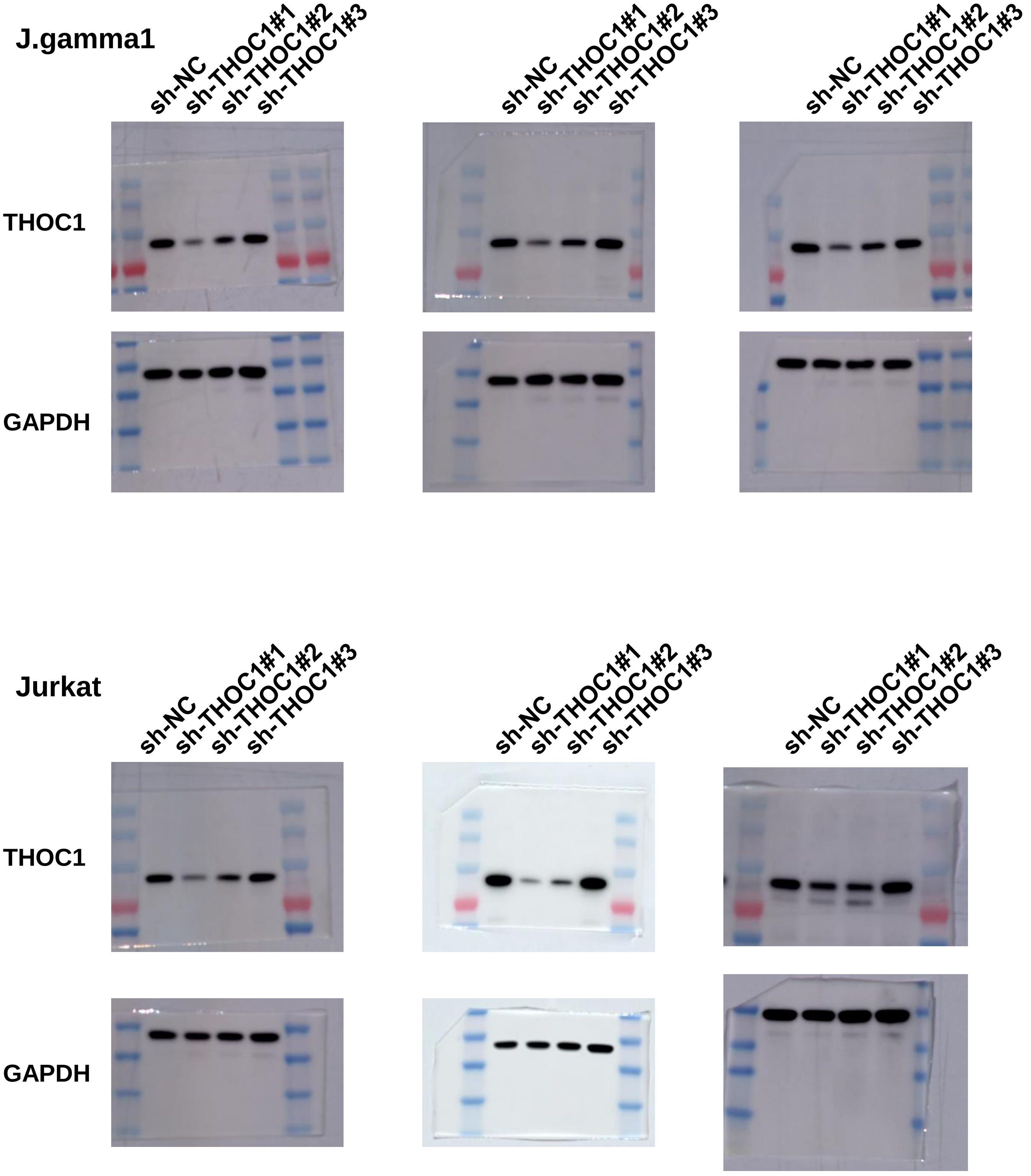
J.gamma1
sh-NC
sh-THOC1#1
sh-THOC1#2
sh-THOC1#3
THOC1
GAPDH
Jurkat
sh-NC
sh-THOC1#1
sh-THOC1#2
sh-THOC1#3
THOC1
GAPDH

Figure 7i

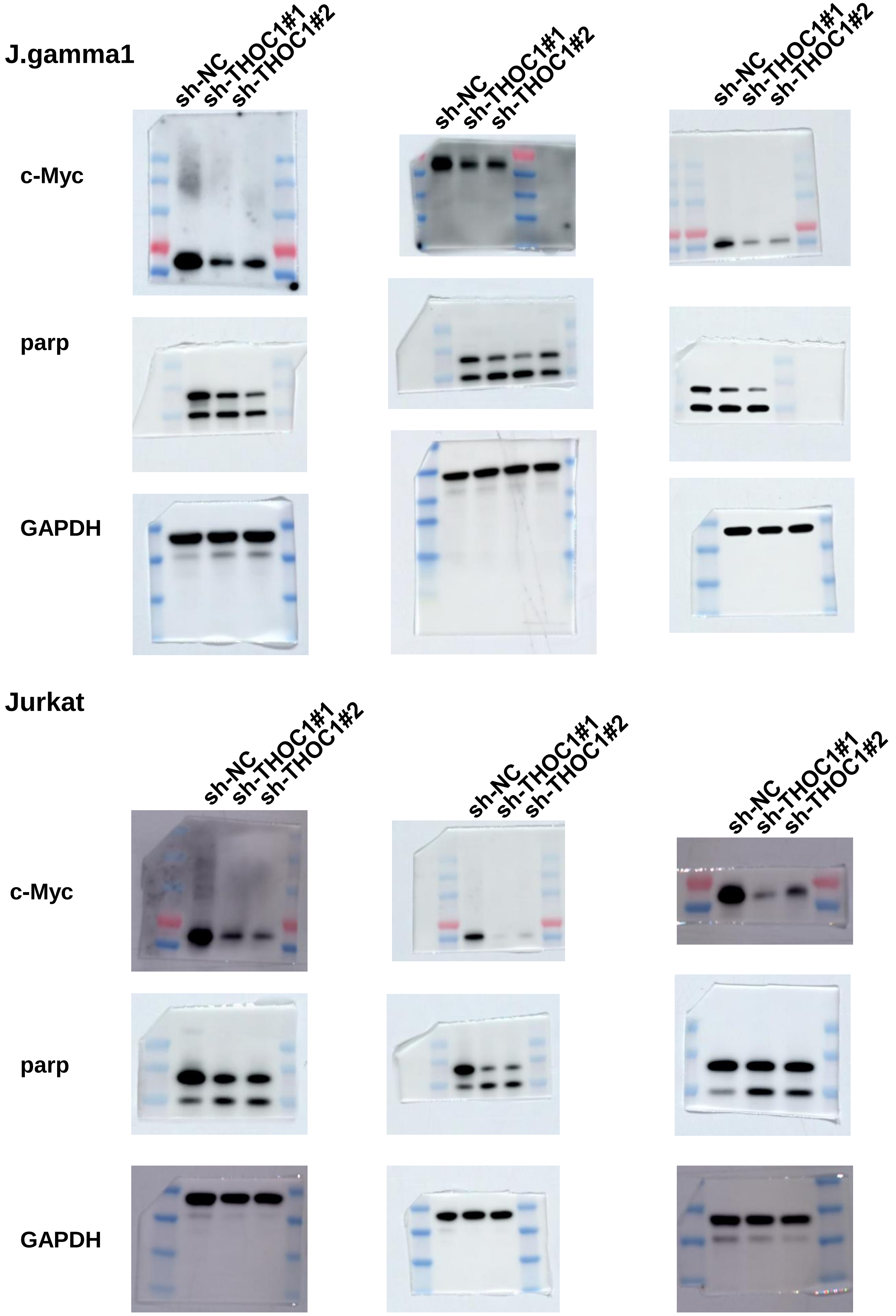
J.gamma1
sh-NC
sh-THOC1#1
sh-THOC1#2
c-Myc
parp
GAPDH
Jurkat
sh-NC
sh-THOC1#1
sh-THOC1#2
c-Myc
parp
GAPDH

Figure 8d

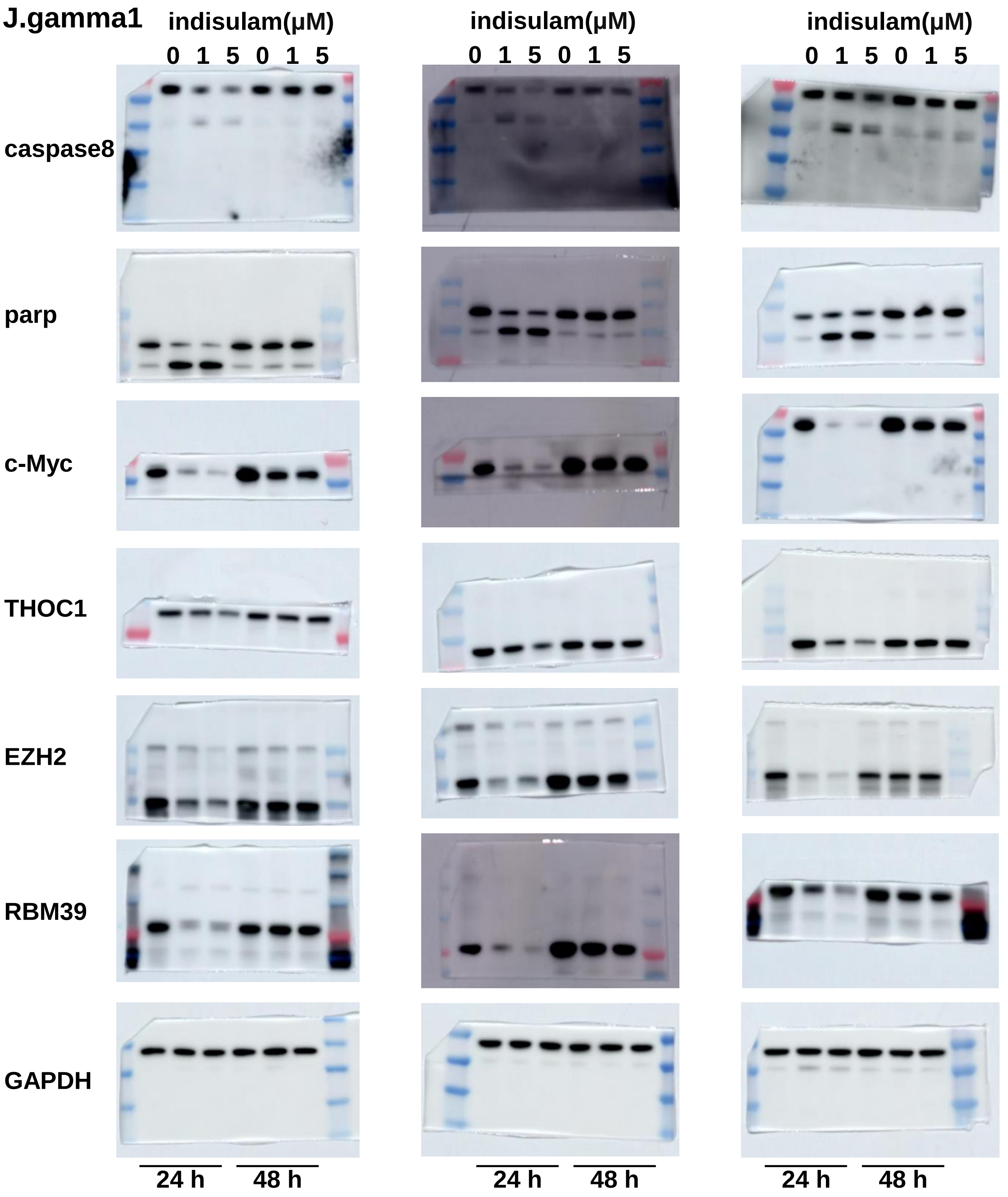
J.gamma1
indisulam(μM)
0 1 5 0 1 5
indisulam(μM)
0 1 5 0 1 5
indisulam(μM)
0 1 5 0 1 5
caspase8
parp
c-Myc
THOC1
EZH2
RBM39
GAPDH
24 h
48 h
24 h
48 h
24 h
48 h

Figure 8d

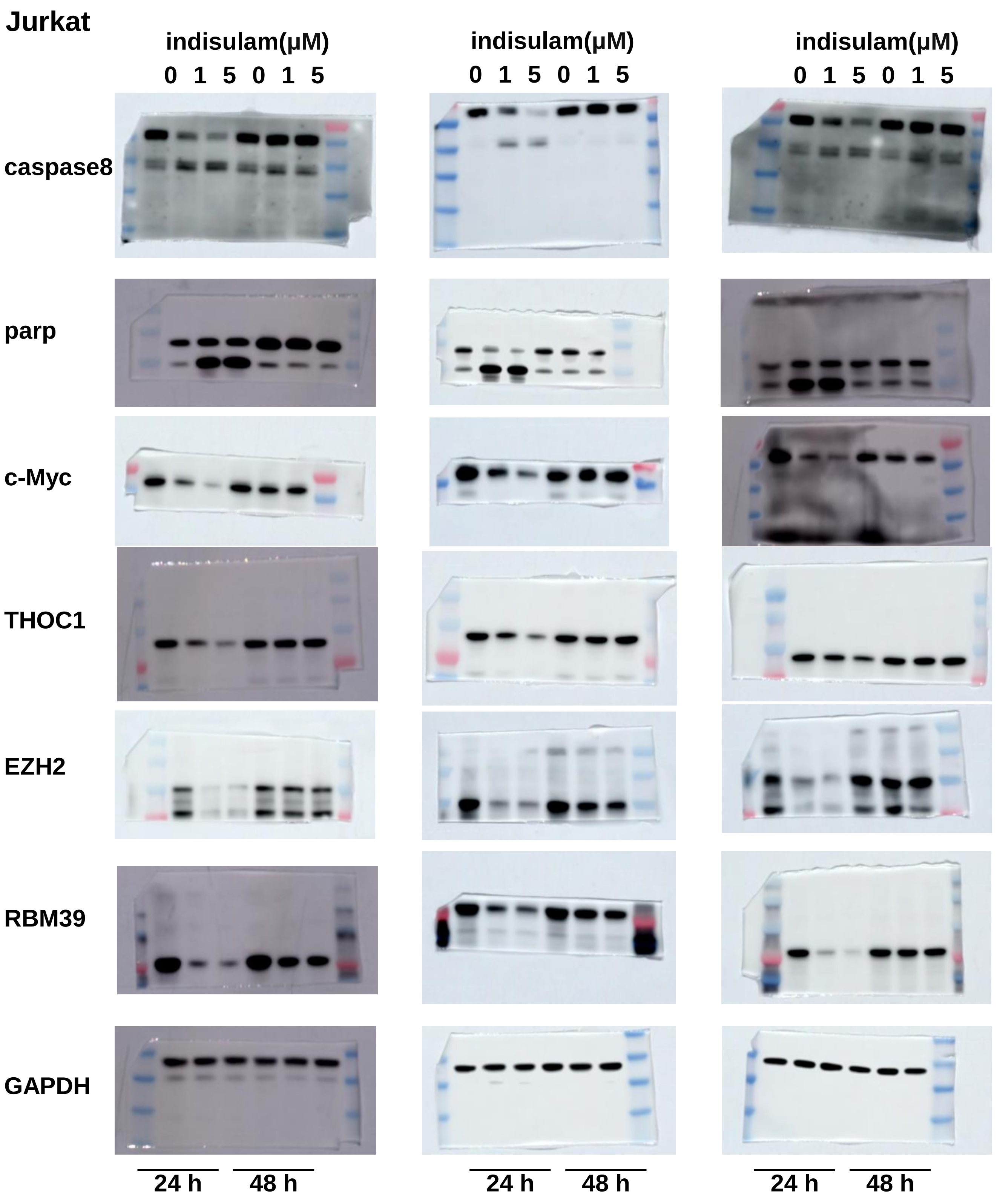

Jurkat
indisulam(μM)
0 1 5 0 1 5
indisulam(μM)
0 1 5 0 1 5
indisulam(μM)
0 1 5 0 1 5
caspase8
parp
c-Myc
THOC1
EZH2
RBM39
GAPDH
24 h
48 h
24 h
48 h
24 h
48 h

Figure 9d

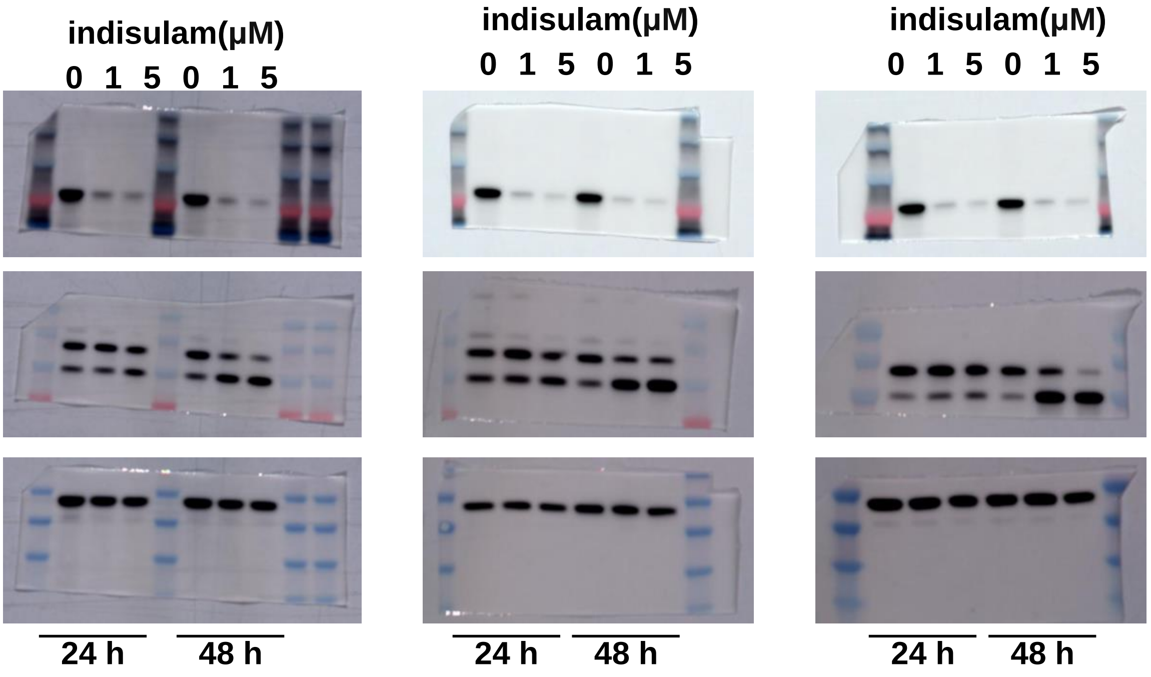

indisulam(μM)
0 1 5 0 1 5
24 h
48 h
indisulam(μM)
0 1 5 0 1 5
24 h
48 h
indisulam(μM)
0 1 5 0 1 5
24 h
48 h

Supplementary Figure 9a

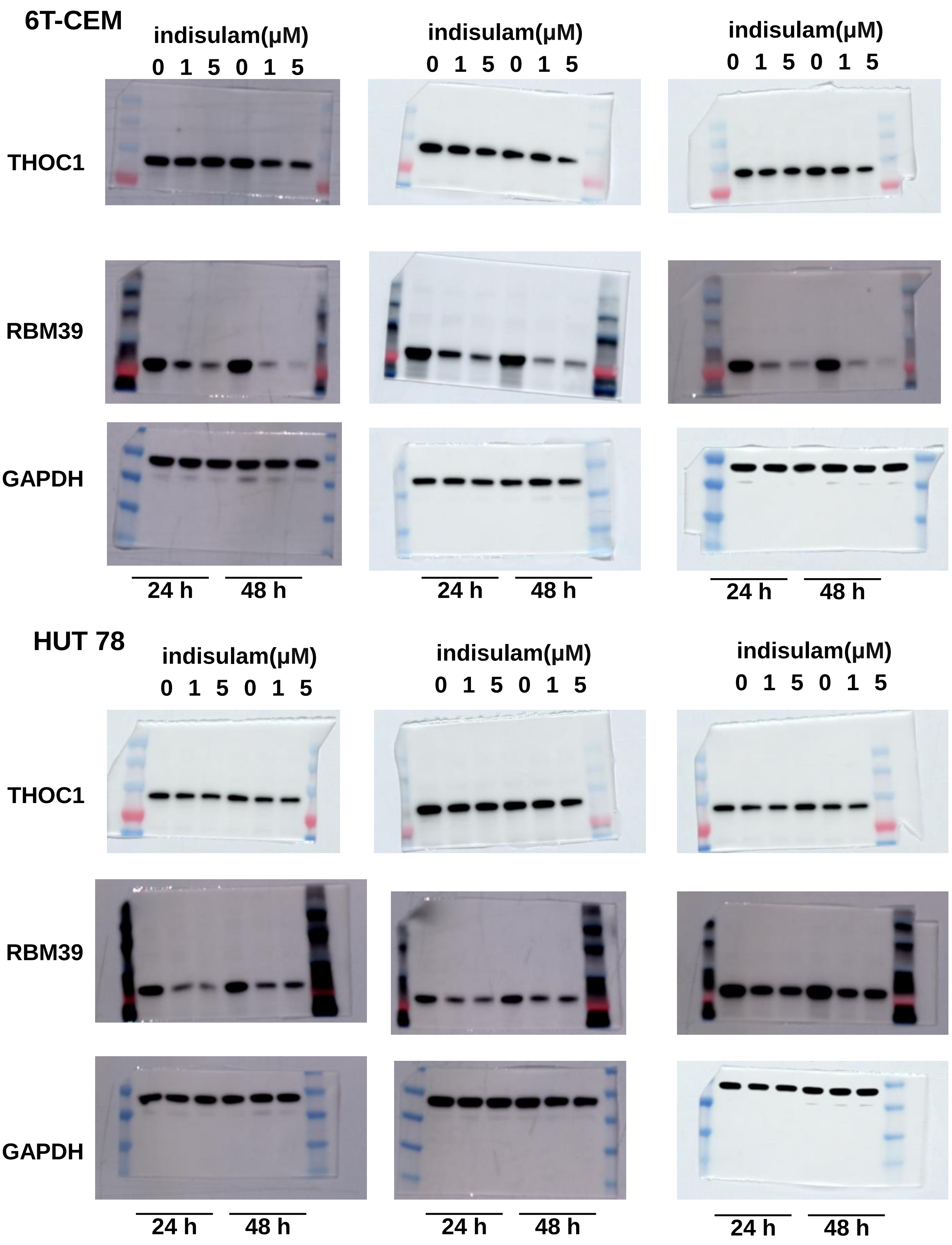

Supplement: Supplementary file 22 — Supplementary Material 22. [file 13046_2024_3130_MOESM22_ESM.pdf]
