# Supplementary material for: Targeting RBM39 through indisulam induced mis-splicing of mRNA to exert anti-cancer effects in T-cell acute lymphoblastic leukemia
Source: J Exp Clin Cancer Res. 2024 Jul 24;43:205. doi: 10.1186/s13046-024-03130-8 (PMC11267830; doi:10.1186/s13046-024-03130-8)
Supplement: Supplementary file 23 — Supplementary Material 23. [file 13046_2024_3130_MOESM23_ESM.pdf]

Cell Line Authentication Report

Cell Line Authentication Report

Customer: Zimu Zhang

Quotation Number: 80-1075874109_R2

Completion Date: 11/30/2022

1. **Sample ID: Jurkat**
2. **Original Material: Cell pellet**
3. **Methods:**

1).Genomic DNA was extracted from the cell pellets provided by the customer.

2).Samples, together with positive and negative control were amplified using GenePrint 10 System (Promega).

3). Amplified products were processed using the ABI3730xl Genetic Analyzer.

4). Data were analyzed using GeneMapper4.0 software and then compared with the ATCC, DSMZ, JCRB and RIKEN databases for reference matching.

4. **Results:**

1) **10 Loci STR Profile：**

Genetic Site	Cell Bank information		Customer sample	
(Locus)	Jurkat		Jurkat	
Amelogenin	X	Y	X	Y
CSF1PO	11	12	11	12
D13S317	8	12	8	12
D16S539	11	11	11	11
D5S818	9	9	9	9
D7S820	8	12	8	12
THO1	6	9.3	6	9.3
TPOX	8	10	8	10
vWA	18	18	18	18
D21S11			31.2	33.2
Percent match between the sample and the database profile：100%				

Summary:

Your cell line is considered to be “identical” to the reference cell line in the Cell Bank STR database, as the STR profile yields a 100% match.

Notes：

1. P=100% x (2xM)/N; M=18, N=36 P=100% x (2x18)/36=100%

M: number of the matching peaks; N: number of all peaks

2. Based on the ANSI Standard, cell lines with ⩾80% match are considered to be related; i.e., derived from a common ancestry. Cell lines with between a 55% to 80% match require further profiling for authentication of relatedness.

3. The short tandem repeat (STR) profile generated by Azenta is indicative only of the sample sent to Azenta at the time it was sent. This data and analysis are for research use only.

2) Electrophoretogram

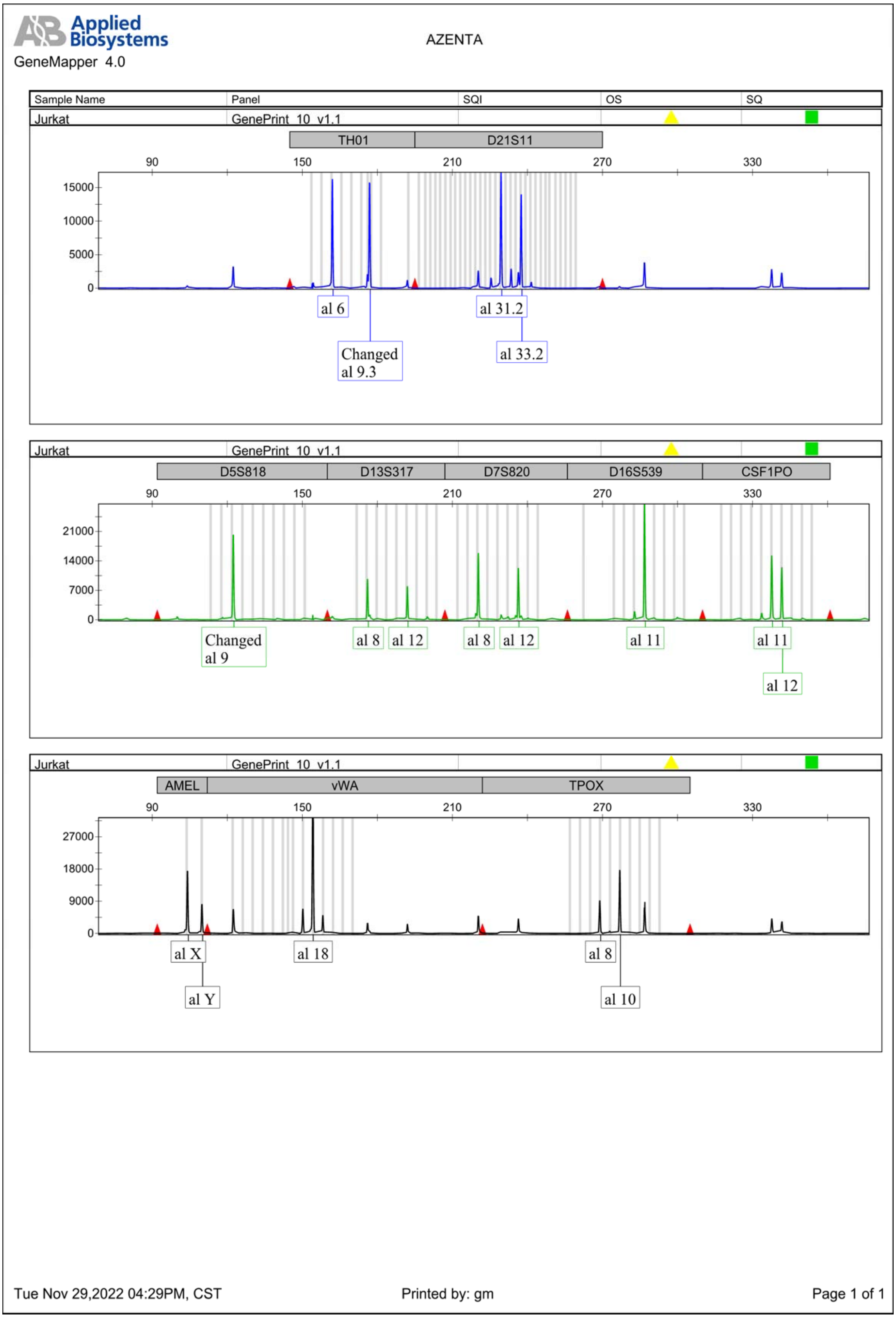

Note：Raw data in appendix

AZENTA
LIFE SCIENCES

Cell Line Authentication Report

Cell Line Authentication Report

Customer: Zimu Zhang

Quotation Number: 80-1057423247_R2

Completion Date: 11/07/2022

1. **Sample ID: 6T-CEM**
2. **Original Material: Cell pellet**
3. **Methods:**

1).Genomic DNA was extracted from the cell pellets provided by the customer.

2).Samples, together with positive and negative control were amplified using GenePrint 10 System (Promega).

3). Amplified products were processed using the ABI3730xl Genetic Analyzer.

4). Data were analyzed using GeneMapper4.0 software and then compared with the ATCC, DSMZ, JCRB and RIKEN databases for reference matching.

4. **Results:**

1) **10 Loci STR Profile：**

Genetic Site	Cell Bank information		Customer sample	
(Locus)	6T-CEM		6T-CEM	
Amelogenin	X	X	X	X
CSF1PO	10	11	10	11
D13S317	11	12	11	12
D16S539	10	13	10	13
D5S818	11	13	11	13
D7S820	9	14	9	14
THO1	6	7	6	7
TPOX	8	8	8	8
vWA	17	19	17	19
D21S11			31	33.2
Percent match between the sample and the database profile：100%				

Summary:

Your cell line is considered to be “identical” to the reference cell line in the Cell Bank STR database, as the STR profile yields a 100% match.

Notes：

1. P=100% x (2xM)/N; M=18, N=36 P=100% x (2x18)/36=100%

M: number of the matching peaks;   N: number of all peaks

2. Based on the ANSI Standard, cell lines with ≥80% match are considered to be related; i.e., derived from a common ancestry. Cell lines with between a 55% to 80% match require further profiling for authentication of relatedness.

3. The short tandem repeat (STR) profile generated by Azenta is indicative only of the sample sent to Azenta at the time it was sent. This data and analysis are for research use only.

2) Electrophoretogram

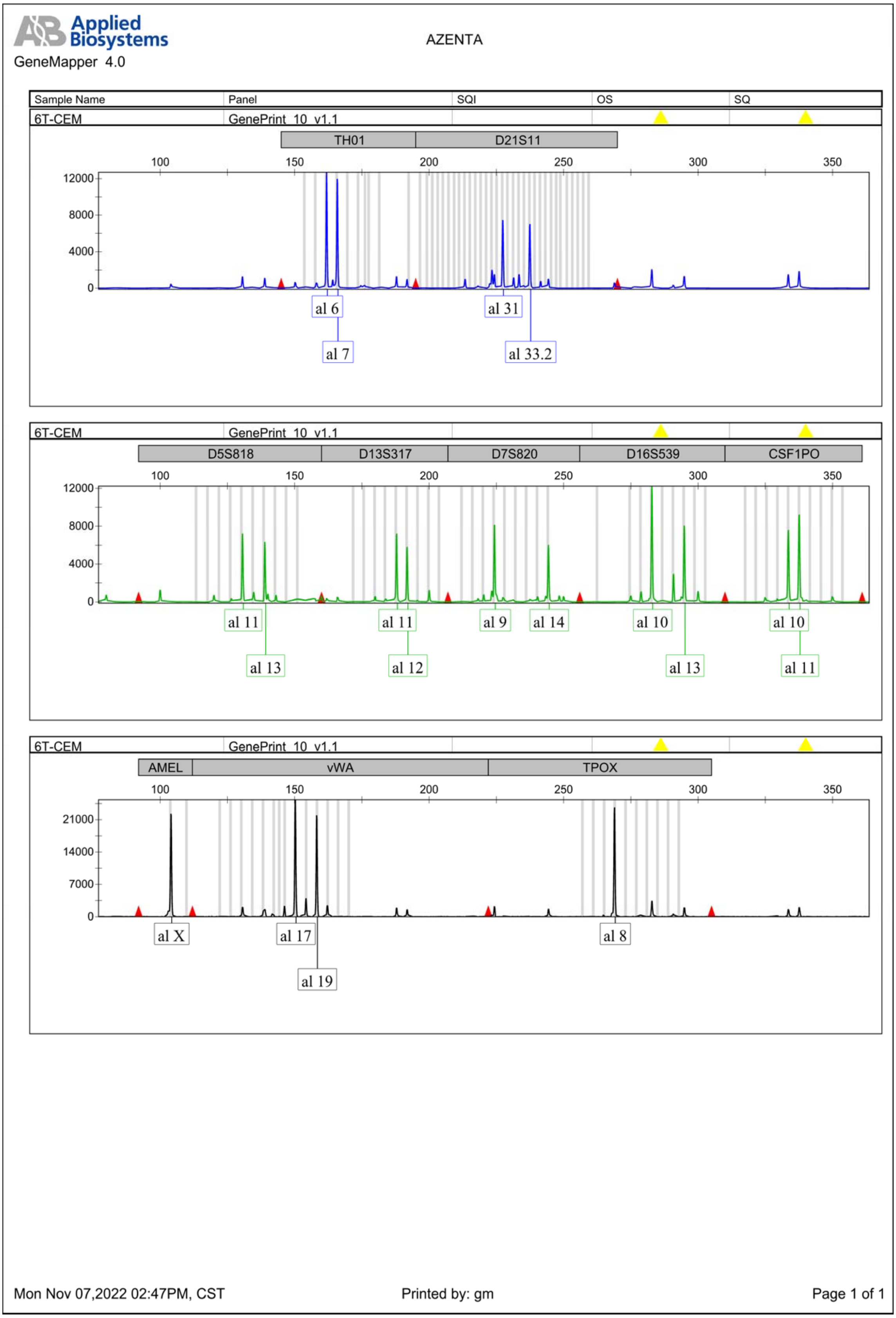

Note：Raw data in appendix

Cell Line Authentication Report

Cell Line Authentication Report

Customer: Zimu Zhang

Quotation Number: 80-1091673806_R2

Completion Date: 12/16/2022

1. **Sample ID: J.gamma1**
2. **Original Material: Cell pellet**
3. **Methods:**

1).Genomic DNA was extracted from the cell pellets provided by the customer.

2).Samples, together with positive and negative control were amplified using GenePrint 10 System (Promega).

3). Amplified products were processed using the ABI3730xl Genetic Analyzer.

4). Data were analyzed using GeneMapper4.0 software and then compared with the ATCC, DSMZ, JCRB and RIKEN databases for reference matching.

4. **Results:**

1) 10 Loci STR Profile：

Genetic Site	Cell Bank information		Customer sample	
(Locus)	J.gamma1		J.gamma1	
Amelogenin	X	X	X	X
CSF1PO	11	12	11	11
D13S317	8	12	8	12
D16S539	11	11	11	11
D5S818	9	9	9	9
D7S820	8	12	8	12
THO1	6	9.3	6	9.3
TPOX	8	10	8	10
vWA	18	19	18	18
D21S11			31.2	33.2
Percent match between the sample and the database profile：88.89%				

Summary:

Your cell line is considered “related” to the reference cell line in the Cell Bank STR database, as the STR profile yields matches that are ≥ 80% but less than 100%.

Notes：

1. P=100% x (2xM)/N; M=16, N=36 P=100% x (2x16)/36=88.89%

M: number of the matching peaks; N: number of all peaks

2. Based on the ANSI Standard, cell lines with ≥80% match are considered to be related; i.e., derived from a common ancestry. Cell lines with between a 55% to 80% match require further profiling for authentication of relatedness.

3. The short tandem repeat (STR) profile generated by Azenta is indicative only of the sample sent to Azenta at the time it was sent. This data and analysis are for research use only.

AZENTA LIFE SCIENCES

2) Electrophoretogram

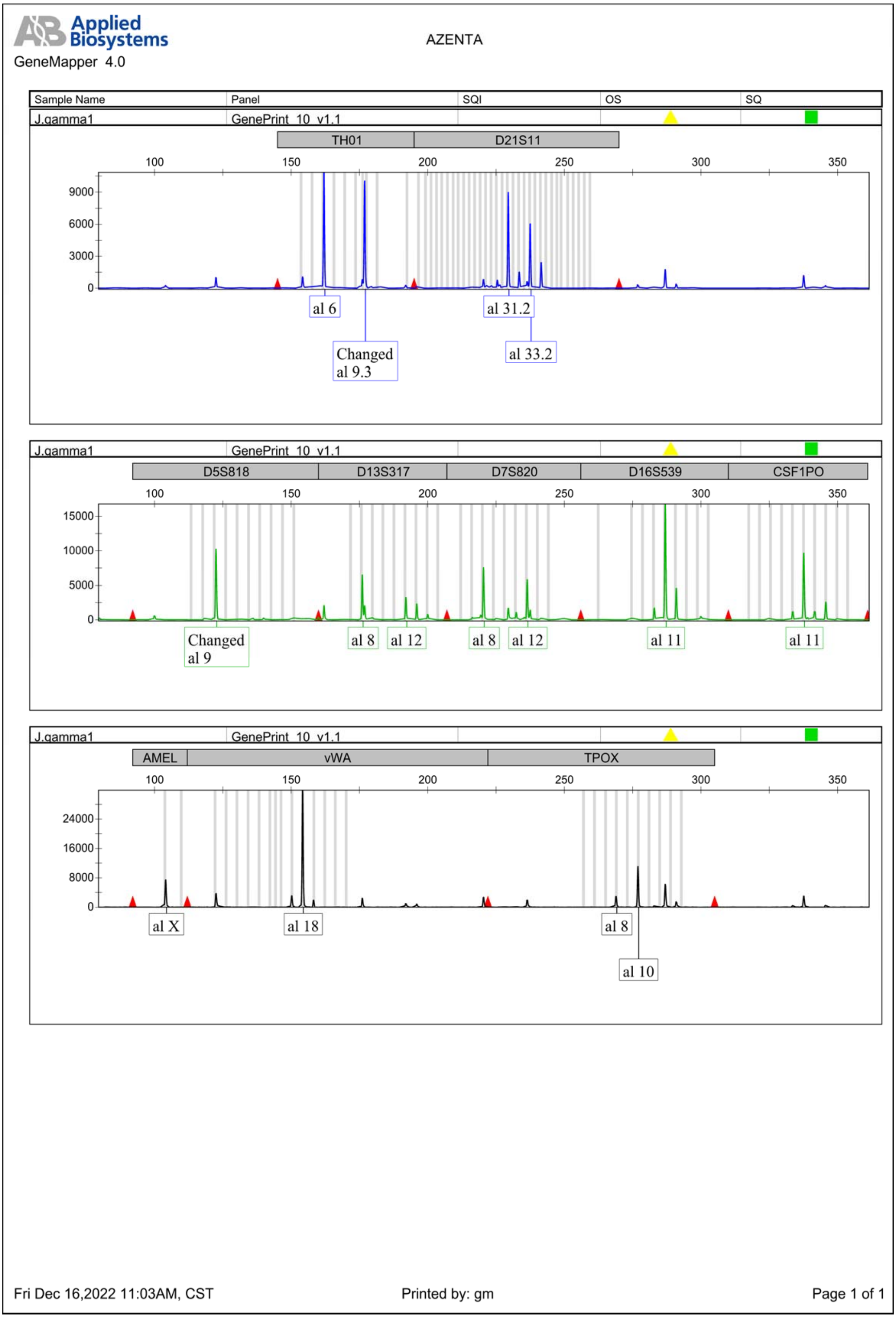

Note：Raw data in appendix

Cell Line Authentication Report

AZENTA LIFE SCIENCES

Cell Line Authentication Report

Customer: Zimu Zhang

Quotation Number: 80-1148132049_R2

Completion Date: 03/16/2023

1. **Sample ID: CCRF**
2. **Original Material: Cell pellet**
3. **Methods:**

1).Genomic DNA was extracted from the cell pellets provided by the customer.

2).Samples, together with positive and negative control were amplified using GenePrint 10 System (Promega).

3). Amplified products were processed using the ABI3730xl Genetic Analyzer.

4). Data were analyzed using GeneMapper4.0 software and then compared with the ATCC, DSMZ, JCRB and RIKEN databases for reference matching.

4. **Results:**

1) 10 Loci STR Profile：

Genetic Site	Customer sample	
(Locus)	CCRF	
Amelogenin	X	X
CSF1PO	11	11
D13S317	11	12
D16S539	10	13
D5S818	12	13
D7S820	9	13
THO1	6	7
TPOX	8	8
vWA	17	19
D21S11	30	33.2

<<< Search for reference matching with the Cell Bank databases and add the match results. >>>

Result of STR matching analysis by your data.
-DSMZ Profile Database-

EV	Cell No.	Cell name	Locus names								
			D5S818	D13S317	D7S820	D16S539	VWA	TH01	AM	TPOX	CSF1PO
	Query(Your Cell)		12,13	11,12	9,13	10,13	17,19	6,7	X,X	8,8	11,11
0.94(34/36)	CCL-119	CCRF-CEM [CCRF CEM]	12,13	11,12	9,13	10,13	17,19	6,7	X,X	8,8	10,11
0.94(34/36)	IFO50412	CCRF-CEM	12,13	11,12	9,13	10,13	17,19	6,7	X,X	8,8	10,11
0.94(34/36)	JCRB0033	CCRF-CEM	12,13	11,12	9,12	10,13	17,19	6,7	X,X	8,8	11,11
0.94(34/36)	JCRB9023	CCRF-CEM	12,13	11,12	9,13	10,13	17,19	6,7	X,X	8,8	10,11
0.89(32/36)	CRL-2264	CEM/C2	13,13	11,12	9,8.3	10,13	17,19	6,7	X,X	8,8	11,11
0.84(32/38)	CRL-8296	6T-CEM	11,12,13	11,12,13	9,14	10,13	17,19	6,7	X,X	8,8	10,11
0.83(30/36)	CRL-8436	CCRF-CEM	12,13	11,11	9,13	10,13	17,19	6,7	X,X	8,8	10,13
0.81(30/37)	CRL-2265	CEM/C1	12,13	9,10,12	9,12	10,13	18,19	6,7	X,X	8,8	11,11
0.81(30/37)	CRL-7916	CCRF-CEM	12,15	11,12	9,13	10,13	17,19,20	6,7	X,X	8,8	10,12
0.78(28/36)	240	CCRF-CEM	12,13	11,11	9,13	10,13	17,19	6,7	X,X	7,8	10,13
0.78(28/36)	CRL-8199	AGR-ON	12,12	11,13	10,13	10,13	17,19	6,7	X,X	8,8	10,11
0.78(28/36)	CRL-8993	800000	12,12	12,13	9,13	10,13	17,20	6,7	X,X	8,8	9,11
0.78(28/36)	TIB-195	CEM-CM3	12,13	12,12	10,13	10,12	18,19	6,7	X,X	8,8	11,11
0.77(30/39)	RCB1980	CCRF-CEM	12,13	10,11,12	9,12	10,13,14	18,19	6,7	X,X	8,8	9,10,11
0.72(26/36)	CRL-2104	CCD-1087Sk	11,12	12,12	9,10	10,13	17,19	7,7	X,X	8,8	11,12
0.72(26/36)	CRL-2510	CCD-1120SK	11,12	11,13	10,12	10,13	17,19	6,9	X,X	8,8	11,11
0.67(24/36)	CRL-2466	CCD-1118SK	12,13	11,12	9,10	11,13	16,17	6,9	X,X	8,11	11,12
0.67(24/36)	RCB1151	RCC10RGB	10,12	11,12	12,13	12,13	14,18	6,9	X,X	8,8	11,11
0.65(26/40)	JCRB1067	CPT-K5	11,12,13	10,12	9,12,14	10,14	17,18,20	6,7	X,X	8,8	9,11,12

Summary

Your cell line is considered “related” to the reference cell line CCRF-CEM [CCRF CEM] in the ATCC STR database, as the STR profile yields matches that are ≥ 80% but less than 100%.

2) Electrophoretogram

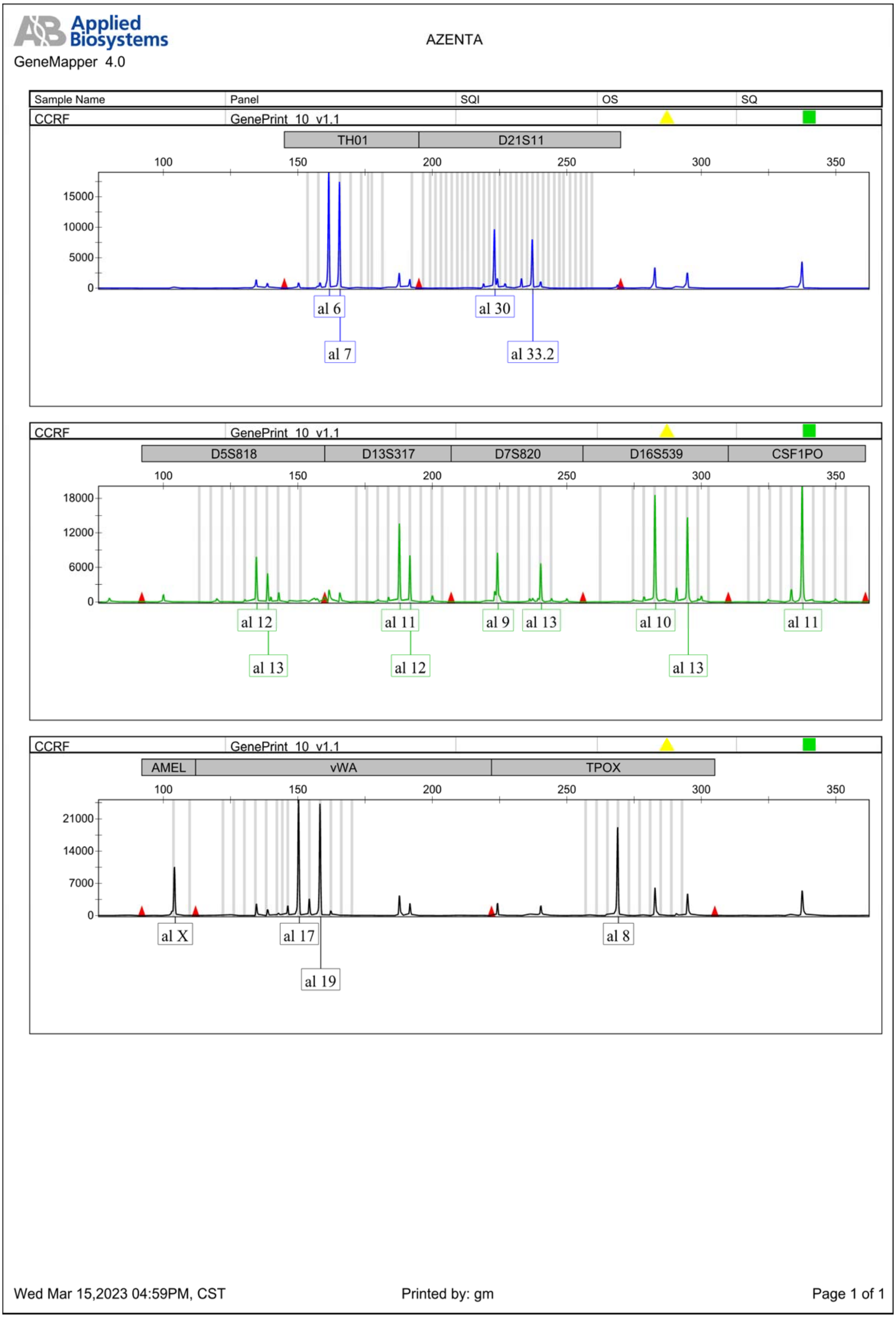

Note：Raw data in appendix
